# Microenvironmental niches dictate divergent fibroblast fates in reversible versus progressive lung fibrosis

**DOI:** 10.1016/j.ebiom.2026.106142

**Published:** 2026-01-30

**Authors:** Licheng Song, Yi Yang, Yifan Fu, Yaru Liu, Qi Li, Mengli Zheng, Chen Yao, Dingyun Song, Ruofan Su, Wen Chen, Jingyu Chen, Huaiyong Chen, Lixin Xie

**Affiliations:** a Senior Department of Respiratory and Critical Care Medicine, The Eighth Medical Center of PLA General Hospital, Beijing, China; b College of Clinical, Chinese Academy of Medical Sciences and Peking Union Medical College, Beijing, China; c Tuberculosis Department, Beijing Chest Hospital, Capital Medical University, Beijing, China; d Wuxi Lung Transplantation Centre, Wuxi People's Hospital Affiliated with Nanjing Medical University, Wuxi, China; e Department of Basic Medicine, Haihe Clinical College, Tianjin Medical University, Tianjin, China; f Key Research Laboratory for Infectious Disease Prevention for State Administration of Traditional Chinese Medicine, Tianjin Institute of Respiratory Diseases, Tianjin, China; g Tianjin Key Laboratory of Lung Regenerative Medicine, Haihe Hospital, Tianjin University, China; h Key Laboratory of Medical Rescue Key Technology and Equipment, Ministry of Emergency Management, Beijing, China

**Keywords:** Organising pneumonia, Idiopathic pulmonary fibrosis, Connective tissue disease-associated interstitial lung disease, Spatial transcriptomics, Glucocorticoid

## Abstract

**Background:**

Fibroblast behaviour is a key determinant of outcomes in interstitial lung diseases (ILDs), yet mechanisms governing the switch between reversible repair and progressive fibrosis remain unclear. How disease-specific cellular niches shape fibroblast fate across ILD phenotypes has not been compared in situ.

**Methods:**

We profiled peripheral lung tissues from controls, organising pneumonia (OP), connective tissue disease–associated ILD (CTD-ILD), and idiopathic pulmonary fibrosis (IPF) using High-Definition Visium spatial transcriptomics. A matched single-cell RNA-seq atlas was integrated via robust cell-type deconvolution to map cellular neighbourhoods. Differential expression and pathway activity were validated by immunofluorescence. Predicted ligand–receptor mechanisms and fibroblast responses were tested *in vitro* under glucocorticoid (GC), TGF-β1, and B-cell/MIF perturbations with receptor blockade.

**Findings:**

Disease-specific niches were tightly coupled to fibroblast states. OP exhibited B-cell–AT2–myofibroblast–enriched niches with high GC responsiveness and apoptosis. IPF was dominated by bronchiolised epithelium, alongside myofibroblasts exhibiting glucocorticoid resistance and strong matrix programmes. CTD-ILD exhibited macrophage-rich niches with multinucleated giant cells. *In vitro*, GC induced NR3C1-mediated apoptosis in fibroblasts, whereas TGF-β1 drove a senescent, GC-resistant phenotype. IgD + B-cell-derived MIF enhanced fibroblast migration via CD74, an effect blunted by TGF-β1. Thus, niche composition dictates fibroblast fate, distinguishing GC-sensitive resolution from apoptosis-resistant fibrosis.

**Interpretation:**

A GC-sensitive, apoptosis-prone myofibroblast niche in OP may underpin reversibility, whereas CTD-ILD and IPF follow distinct trajectories driven by immune dysregulation and epithelial–stromal maladaptation. These spatial microenvironmental signatures nominate therapeutic targets and may inform precision therapy for fibrotic lung disease.

**Funding:**

The 10.13039/501100001809National Natural Science Foundation of China (82172109 to L.X., 82570001 to H.C.), the 10.13039/501100002855Ministry of Science and Technology of the People's Republic of China (2023YFC3502605, 2024YFA1108906) (H.C.), the 10.13039/501100006606Natural Science Foundation of Tianjin, China (25JCZDJC01260) (H.C.); the Key Laboratory of Medical Rescue Key Technology and Equipment, 10.13039/100020763Ministry of Emergency Management (Open Fund Project No. YJBKFKT202410).

**Research in context**

**Evidence before this study**

Across interstitial lung diseases (ILDs), the determinants of reversible repair versus inexorable fibrosis remain unclear in human tissue. Existing single-cell and spatial studies typically examined single ILD entities and seldom linked in situ immune–epithelial neighbourhoods to fibroblast glucocorticoid (GC) responsiveness, apoptosis, and matrix programmes across phenotypes. Critically, despite organising pneumonia (OP) being a GC-responsive ILD with documented clinical reversibility, its fibroblast states and immune microenvironment have not been systematically leveraged as a model to illuminate mechanisms that might be harnessed to induce remission in progressive fibrotic diseases such as idiopathic pulmonary fibrosis (IPF) and CTD-ILD.

**Added value of this study**

We provide a harmonised, High-Definition spatial framework aligned with single-cell references to resolve disease-specific niches and their control of fibroblast fate. By positioning OP as a paradigmatic, GC-responsive condition, we show how a B cell– and AT2-influenced niche selects GC-sensitive, apoptosis-prone myofibroblast states, in contrast to macrophage-dominant CTD-ILD and bronchiolised epithelial niches in IPF that associate with GC hyporesponsiveness, excessive ECM deposition and apoptosis-resistant programmes. Mechanistically, we validate that B cell–derived MIF–CD74 signalling enhances migration of GC-sensitive fibroblasts, while TGF-β1 drives a senescence-like state characterised by blunted GC responses and heightened remodelling activity. Thus, OP offers a tractable in situ model linking spatial ecology to pro-resolution fibroblast behaviour, generating translatable axes relevant beyond OP.

**Implications of all the available evidence**

Spatially defined cellular ecosystems—rather than fibroblast–intrinsic properties alone—govern fibrotic trajectories across ILDs. Therapeutic strategies that reprogramme myofibroblast glucocorticoid sensitivity and pro-apoptotic capacity may potentially promote remission or reversal in otherwise progressive fibrosis. In OP, the B cell–derived MIF–CD74 axis emerges as a plausible driver of GC-sensitive myofibroblast recruitment into alveoli, suggesting a plausible mechanism for lesion formation and a potential therapeutic target. More broadly, discovering and classifying immune–epithelial–mesenchymal niches could help identify fibrosis subtypes with inherent potential for improvement and guide precision interventions.

## Introduction

The divergent clinical trajectories of fibrotic interstitial lung diseases (fILDs) present a fundamental challenge. While idiopathic pulmonary fibrosis (IPF) progresses relentlessly, organising pneumonia (OP) can resolve completely. OP is histopathologically defined by intra-alveolar connective tissue plugs. Its aetiology ranges from autoimmune and drug-related triggers^1^^,^^2^ to cryptogenic forms potentially linked to undetected antigens.^3^ The relevance of this process is underscored by the emergence of OP patterns in post-COVID-19 lung remodelling.^4^

The reversibility of OP, typically in response to glucocorticoid (GC) therapy, contrasts with the progressive nature of IPF and connective tissue disease-associated ILD (CTD-ILD). Mechanistically, OP resolution involves preserved alveolar basement membranes^5^ and an imbalance favouring matrix metalloproteinases and enhanced fibroblast apoptosis.^6^^,^^7^ While GCs induce rapid radiographic resolution,^8^ the precise spatial determinants governing this therapeutic sensitivity remain undefined. Elucidating the molecular basis of GC reactivity in OP may unveil strategies for intractable diseases like IPF.

Accumulating evidence implicates B-lymphocytes as critical modulators of the fILD microenvironment. In IPF, the accumulation of IgA + B-cells is associated with poor prognosis,^9^^,^^10^^,^^11^^,^^12^^,^^13^^,^^14^^,^^15^ raising questions about their potential role in driving lung injury. This is further supported by the abundance of IgG autoantibodies in UIP lungs^16^ and their correlation with decreased pulmonary function.^17^ Beyond antibody production, B-cells may function as inflammatory mediators that contribute to ILD exacerbation,^18^ while IgM + anti-myeloperoxidase B-cells have been shown to exacerbate vasculitis-associated ILD via complement activation.^19^ Despite these associations, how the B-cell niche specifically influences fibroblast behaviour and contributes to the divergent outcomes between resolving and progressive fibrosis remains poorly understood.

Current therapeutic strategies targeting terminal fibroblast activation in IPF have yielded limited success, suggesting the myofibroblast is not a monolithic entity but a plastic cell state governed by its microenvironment. We propose that the opposing trajectories of OP and IPF offer a unique opportunity to dissect the regulatory networks determining fibrotic resolution versus persistence. In this study, we integrated High-Definition spatial profiling with single-cell RNA sequencing to map cellular ecosystems in OP, CTD-ILD, and IPF, aiming to define the distinct functional states of myofibroblasts and the niche-specific cues that dictate their fate.

## Methods

A detailed description is provided in the Supplementary methods.

### Ethics and samples

Human lung samples (Control, CTD-ILD, OP, IPF) were obtained with the approval of the Ethics Committee of the Chinese PLA General Hospital (#2022052701006). CTD-ILD and IPF tissues were obtained from lung transplant recipients diagnosed with CTD-ILD and bilateral pulmonary fibrosis, respectively. OP tissues were collected from surgical lobectomies of patients with postoperative pathological confirmation of OP. Control tissues were obtained from deceased donors without lung disease. In addition, to address potential resection bias, we assembled an independent biopsy-plus-steroids OP cohort (n = 10) and conducted pre-specified pattern-level equivalence testing against the surgical OP cohort (n = 10). All participants were Chinese. Sex assigned at birth was abstracted from the medical record as female or male. No inclusion or exclusion criteria were applied based on sex or ethnicity. Sample availability and tissue quality determined enrolment for each data modality. Written informed consent was obtained from all participants. Clinical details are provided in Supplementary Tables S1 and S2, and in Supplementary Methods “Sample Preparation of Human Lung Tissues”.

### Standardised H&E histopathology annotation, quantification, and staging for OP

Standardised haematoxylin and eosin (H&E) histopathology was performed to annotate representative lung contexts (including blood vessels, airways, and fibrotic lesions) for High-Definition (HD) Visium selection. For OP, we implemented an objective ROI-level staging system (early inflammatory, middle fibro-inflammatory, and late fibrotic) based on intra-alveolar filling patterns and cellular composition. Quantification was performed using supervised segmentation with blinded pathologist review. Detailed staging criteria and reproducibility metrics are described in the Supplementary Methods.

### Single-cell RNA sequencing and analysis

OP is diagnosed post hoc; rapid glucocorticoid response makes obtaining fresh OP tissue for scRNA-seq infeasible and ethically unjustified. Therefore, we used non-OP references (CTRL, CTD-ILD, IPF) to deconvolve HD Visium, leveraging conserved lung lineages and OP-like pathological features present in CTD-ILD and specific IPF niches. Single-cell suspensions of fresh lung tissues from four control donors, three patients with CTD-ILD, and five patients with IPF were prepared via enzymatic digestion and processed using the 10× Genomics platform. After quality control and doublet removal, data were integrated to define cell types. This non-OP reference was used to deconvolve HD Visium data, leveraging conserved lung lineages and OP-like pathological features present in other fILDs. Trajectory analysis (Slingshot) and cell–cell communication (CellChat) were performed on fibroblast and immune subsets.

### Spatial transcriptomics (HD visium)

We performed high-resolution spatial transcriptomics on formalin-fixed paraffin-embedded (FFPE) lung sections from controls (5 subjects) and patients with CTD-ILD (5 subjects), OP (6 subjects), or IPF (5 subjects). FFPE sections (DV200 > 30%) were subjected to H&E staining, pathological annotation of lesions, and HD Visium processing (CG000684/685; 10× Genomics; 10xgenomics.com). Probes were hybridised, ligated, and captured using CytAssist. Libraries were sequenced using Illumina NovaSeq X Plus. Data alignment (Space Ranger v3.0.0; GRCh38 reference) and spot selection were guided by histopathological features (Control: Alveoli, bronchioles, and vessels; CTD-ILD: Diffuse lung injury and interstitial thickening regions; OP: Intra-alveolar fibrotic plugs; IPF: Fibroblast foci and bronchiolisation). Sixty-three ROIs (three per sample) were extracted for downstream analysis.

### Spatial data integration and microenvironmental analysis

Cell-type distributions were mapped via robust cell-type decomposition (RCTD) using the integrated scRNA-seq reference. Spatial interactions were inferred based on Euclidean distances between cell-type centroids. Gene signature scores (apoptosis, GC response, inflammation, B-cell differentiation, collagen biosynthesis) were computed using UCell. To define “GC-sensitive” cells, we applied a per-sample threshold (top 25th percentile of GC response score) to ensure cross-sample comparability. Sensitivity analyses using alternative thresholds and bootstrap estimation are detailed in the Supplementary Methods.

### Immunofluorescence (IF) and antibody validation

Multiplex immunofluorescence (IF) was performed on FFPE sections to validate spatial colocalisation of key markers (including IgJ, IgD, CD19, CD68, CD74, Macrophage Migration Inhibitory Factor [MIF], Glucocorticoid Receptor [NR3C1], Lactotransferrin [LTF], Cytokeratin 5 [KRT5], cytokeratin 17 [KRT17], SFTPC, SCGB1A1/CC10, ACTA2, Fibronectin, and Collagen I). Apoptosis markers included Caspase-3 and Cleaved PARP1. Detailed information regarding antibody clones, RRIDs, and validation is provided in Supplementary Table S3.

### *In vitro* functional validation

For functional validation, human B-cells were isolated from peripheral blood and co-cultured with MRC-5 human foetal lung fibroblasts. Fibroblasts were stimulated with TGF-β1 or dexamethasone, or exposed to conditioned media from resting or activated B-cells. The roles of the MIF–CD74 axis were assessed using the MIF inhibitor MIF098 and the anti-CD74 antibody Milatuzumab. Fibroblast migration was quantified by scratch-wound assays, and protein expression was analysed via Western blotting. Further details are provided in the Supplementary Methods.

### Statistical analysis

All statistical analyses were performed using R software (version 4.3.0; https://www.r-project.org/) and GraphPad Prism (version 9.0; San Diego, CA, USA). Statistical methods for each analysis are detailed in the Methods section. No statistical methods were used to predetermine sample size. Human samples were maximised based on availability, tissue quality, cost, and throughput, and the number of donors was aligned with similar spatial transcriptomics studies. Experiments were not randomised. Between-group differences in proportions, distances, and module scores were tested at the donor level using Kruskal–Wallis with BH-adjusted pairwise tests (Dunn or Wilcoxon as appropriate), alongside effect sizes and bootstrapped CI. Sex information reflects the clinical record and was used in descriptive cohort characterisation and sensitivity checks; the spatial transcriptomic analyses and mechanistic inferences were not stratified by sex due to limited sample size within each disease category and the primary focus on microenvironmental niche comparisons across ILD phenotypes.

### Role of funders

Funders of the study had no role in study design, data collection, data analyses, interpretation, or writing of report.

## Results

### Spatial transcriptomics reveals divergent pathological ecosystems in reversible and progressive fibrosis

**Fig. 1:**
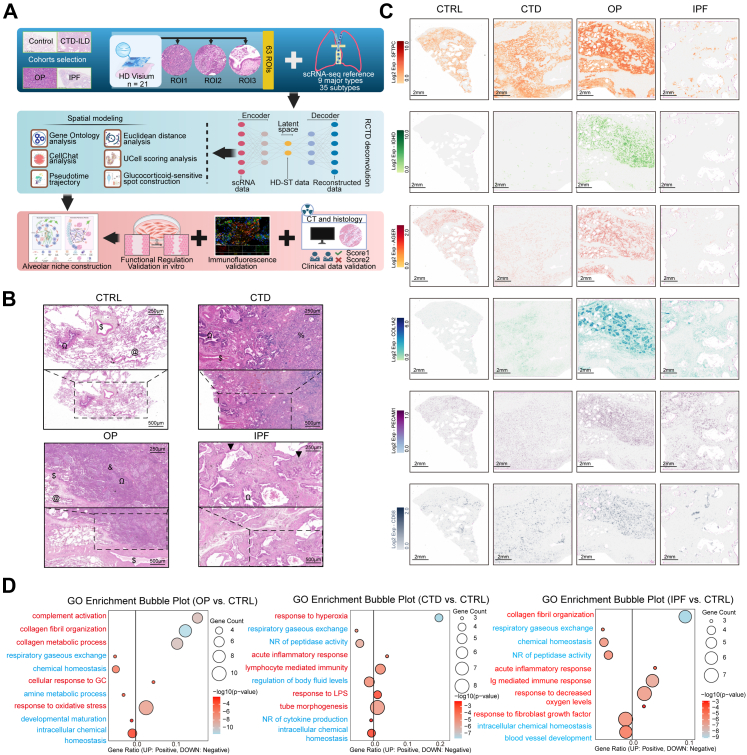
Spatial transcriptomic (ST) profiling reveals fibrotic interstitial lung disease (fILD)-specific molecular signatures. A) Schematic workflow of spatial transcriptomics analysis in fILDs in this study. B) Representative histological regions in controls (CTRL) and three fILD subtypes: organising pneumonia (OP), connective tissue disease-associated ILD (CTD-ILD), and idiopathic pulmonary fibrosis (IPF). Key: @ alveolar space; $ blood vessel; Ω large airway; % diffuse alveolar damage; & intraluminal fibroblastic plugs; ▲ fibroblastic foci. Scale bars, 500 μm and 250 μm (magnified image). C) Expressions of representative gene markers of AT1, AT2, endothelium, fibroblast, macrophage and B cells in ST. Scale bar, 2 mm. D) Dot plots displaying enriched Gene Ontology (GO) terms from pseudobulk differential gene expression analysis comparing CTRLs versus OP, CTD-ILD, and IPF groups (Benjamini–Hochberg [BH]–adjusted q < 0.05).

**Supplementary Figure S1:**
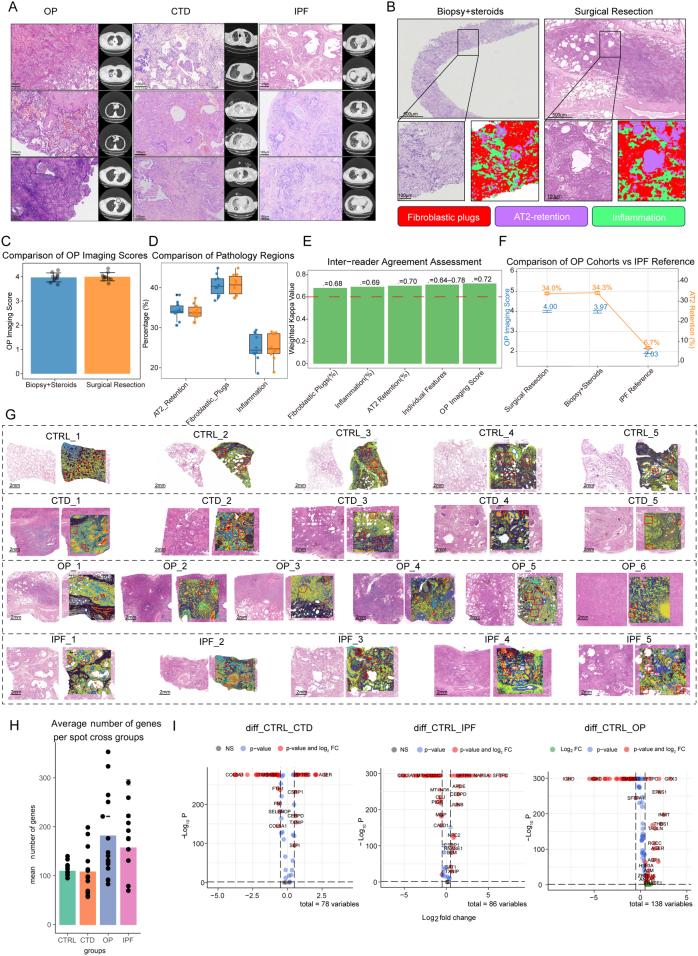
Pathological-radiological correlation in enrolled patients and quality control of spatial transcriptomics. A) Haematoxylin and eosin (H&E)-stained sections of lung tissue samples from patients with organising pneumonia (OP), connective tissue disease-associated interstitial lung disease (CTD-ILD), and idiopathic pulmonary fibrosis (IPF), with corresponding computed tomography (CT) imaging. Black circles on the CT scans indicate the tissue sampling sites for histopathological examination. Scale bar, 100 μm. B) Standardised H&E OP histopathology biopsy (left) vs surgical resection (right) with supervised segmentation of Fibroblastic plugs, AT2-retention, and Inflammation. Scale bars, 100 μm and 500 μm. C) Double-blinded radiology scoring reveals no difference in composite OP Imaging Score (0–6) between biopsy-plus-steroids and surgical OP cohorts (Mann–Whitney U test). Median and interquartile range (IQR; [Q3−Q1]), *P* > 0.05, n = 10 per group. D) Key histopathology metrics proportions are similar between biopsy-plus-steroids and surgical resection OP cohorts, with no statistically significant differences (Mann–Whitney U test). Median and interquartile range (IQR; [Q3−Q1]), *P* > 0.05, n = 10 per group. E) Weighted kappa values met the pre-registered acceptability threshold (κ ≥ 0.60) for radiology features and pathology scores, indicating good inter-reader reliability across domains. F) Both OP cohorts show higher OP Imaging Scores and greater AT2 retention than IPF, supporting OP-specific radiologic–pathologic patterns (Mann–Whitney U test), Median and interquartile range (IQR; [Q3−Q1]), n = 10 per OP group and n = 5 in IPF group. G) H&E staining and spatial distribution of different clusters for spots throughout tissues in the HD Visium capture slides from CTRL, OP, CTD-ILD, and IPF specimens. The region of interest (ROI) of all high-definition (HD) visible samples are annotated with red rectangular boxes. Scale bar, 2 mm. H) Average number of unique genes detected per spot and sample. I) Pseudobulk differential expression analysis comparing pooled CTRL with CTD-ILD, IPF, and OP HD visible data per area to identify significant differentially expressed genes between conditions based on data from entire tissue sections.

Based on representative CT images and correlated H&E sections (Supplementary Figure S1A), we selected three characteristic pathological representative regions of interest (ROIs) from FFPE sections from individuals without lung disease (Control, 5 participants) and patients with CTD-ILD (5 participants), OP (6 participants), and IPF (5 participants, Fig. 1A, Supplementary Figure S1A). To mitigate potential clinical bias between percutaneous biopsy–confirmed OP cases and surgically resected OP cases, we conducted an independent, double-blinded clinical analysis. Surgical OP cases and biopsy-plus-steroids OP cases showed no statistically significant differences in clinical baseline characteristics (Supplementary Table S2) or in CT-based OP Imaging Score and key histopathology metrics (Fibroblastic Plugs, AT2 retention, and Inflammation; all *P* > 0.05), with good inter-reader reliability (Supplementary Figure S1B–F). These cohort-level comparisons indicate that the radiologic–pathologic features analysed here are representative of OP broadly, supporting the generalisability of our spatial findings to the wider OP population. Specimens were processed with haematoxylin and eosin (H&E) staining, and spots captured by 10× HD Visium (Supplementary Figure S1G). Spatial transcriptomic data were generated across 436,003 spots, with the mean number of genes per spot exceeded 100 (Supplementary Figure S1H). Pathological evaluation revealed disease-specific architectural alterations (Fig. 1B). CTD-ILD exhibited diffuse interstitial thickening and alveolar collapse with macrophage accumulation. OP maintained alveolar integrity but showed intra-alveolar infiltration of immune cells and fibroblasts. IPF displayed characteristic interstitial pneumonia (UIP) patterns, including bronchiolisation and fibroblast foci. Compared with control lungs, CTD-ILD lungs preserved SFTPC expression, showed reduced expression of AGER, and increased expression of COL1A2 and the macrophage marker CD68 (Fig. 1C). IPF lungs exhibited increased expression of COL1A2 and PECAM1, along with decreased expression of SFTPC and AGER, indicating enhanced fibrotic activity, vascular remodelling, and loss of alveolar epithelial structure. In contrast, all these markers were elevated in OP lungs, along with increased expression of IGHD. Comprehensive transcriptomic comparisons and GO analysis revealed distinct pathways associated with OP, CTD-ILD, and IPF (Fig. 1D, Supplementary Figure S1I). OP lungs showed upregulation of the GC response and collagen remodelling pathways. CTD-ILD lungs were enriched for hyperoxia and acute inflammation markers, whereas IPF lungs were characterised by predominant extracellular matrix (ECM) deposition and fibroblast signalling. Collectively, while all these fILDs involve injury repair programs, OP is uniquely characterised by GC-responsive activity within preserved alveoli, in contrast to irreversible ECM remodelling of IPF and inflammation-driven pathology of CTD-ILD.

### High- definition (HD) visium spot annotation through cell-type deconvolution

**Fig. 2:**
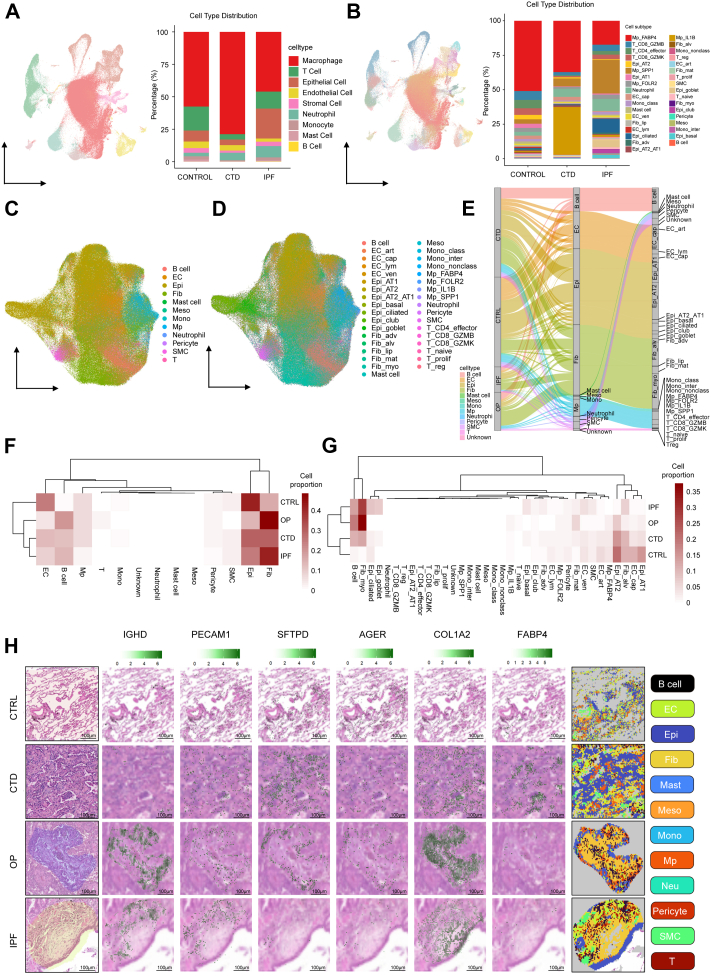
Single-cell RNA sequencing (scRNA-seq) facilitates High-Definition Visium spot annotation via cell-type deconvolution. A) Uniform manifold approximation and projection (UMAP) of major cell types from scRNA-seq of control (CTRL), connective tissue disease-associated interstitial lung disease (CTD-ILD), and idiopathic pulmonary fibrosis (IPF) lungs. Bar plots showing the proportions of different cell types across indicated groups (right). B) UMAP of cell subtypes across disease groups. Bar plots showing the proportions of different cell types across indicated groups (right). C-D) UMAP projections and proportions of major cell types (C) and subtypes (D) from spatial transcriptomic (ST). Bar plots showing the proportions of different cell types across indicated groups. E) Sankey diagram linking cell populations to disease states. F-G) Heatmaps of unsupervised clustering based on major cell-type (F) and subtype (G) proportion matrices. H) Spatial projections of marker genes for major cell types across representative regions of interest (ROIs) in ST profiling, with the pathological images of each group as the background. RCTD assignments showed high-confidence classifications for the majority of spots and strong agreement with canonical marker distributions, consistent with pre-specified quality thresholds. Scale bar, 100 μm.

**Supplementary Figure S2:**
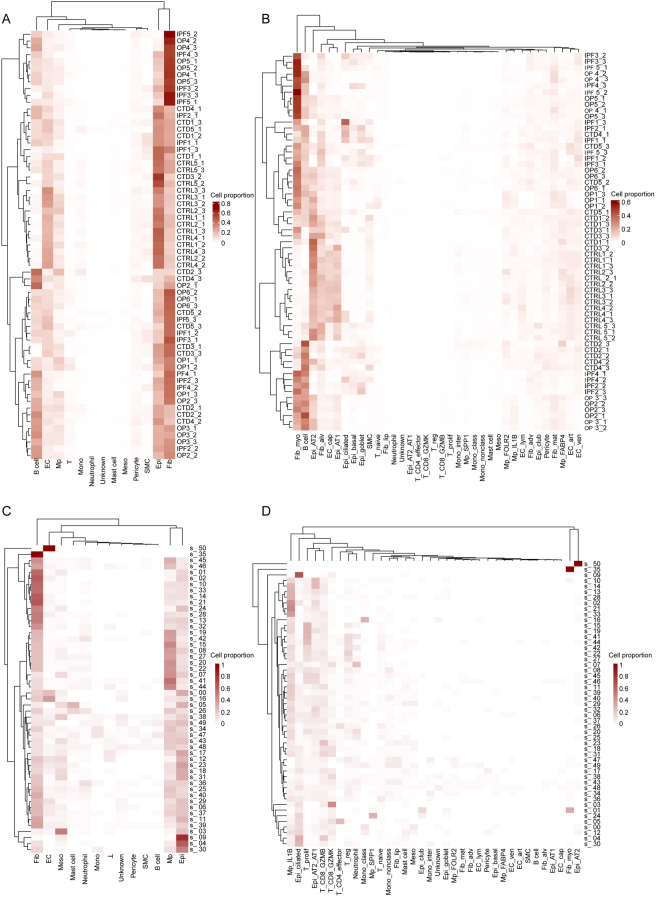
Robust cell-type decomposition (RCTD) integrates single-cell RNA sequencing (scRNA-seq) data with spatial transcriptomic profiles for cell-type–based clustering based on cell-type proportion matrices. A) Unsupervised sample clustering based on major cell-type proportion matrices. B) Unsupervised sample clustering based on cell subtypes proportion matrices. C) Unsupervised clustering based on major cell-type proportion matrices. D) Unsupervised clustering based on cell subtypes proportion matrices.

Complementary single-cell RNA sequencing (scRNA-seq) on fresh lung tissues from four control donors, three patients with CTD-ILD, and five patients with IPF generated a reference atlas of nine major cell types (Fig. 2A) and 35 subtypes (Fig. 2B) through established lung signatures. These included four macrophages, three monocytes, six T-cells, eight fibroblasts, six epithelial cells, and four endothelial subpopulations defined by canonical marker genes. RCTD deconvolution using scRNA-seq reference yielded high-confidence spot assignments (436,003 spatial spots). After quality control, only 207 spots (0.04% of all bins) were annotated as “unknown”, supporting the robustness of downstream spatial inferences (Fig. 2C and D, Supplementary Figure S2A–D). Sankey analysis confirmed high concordance between the original annotations and deconvoluted subtypes (Fig. 2E). Heatmap clustering analysis revealed a predominance of fibroblast-spots and B cell-spots in OP lungs, in contrast to control lungs, which were enriched in epithelial and endothelial cell-spots. IPF lungs exhibited a similar pattern to OP lungs, although with reduced B-cell-spot abundance. Notably, fibrotic lesions in IPF were uniquely enriched adjacent to the bronchial epithelial-spots. In contrast, CTD-ILD lungs showed fewer fibroblast-spots and B-cell-spots and a relative increase in epithelial cell-spots compared with that in OP and IPF lungs (Fig. 2F and G). Biomarkers for subtypes were mapped to histological localisations (Fig. 2H). In lungs with OP, fibroblast-spots form large aggregates with interspersed B-cell-spots in alveolar regions. In IPF lungs, B-cell-spots surrounded fibrotic lesions, whereas in CTD-ILD lungs, fibroblast-spots were distributed diffusely within the alveolar epithelial-spots. This integrated scRNA-seq and High-Definition spatial transcriptomic mapping analysis revealed disease-specific cellular signatures across three fILD pathologies.

### Myofibroblasts are central to fibrotic lesions but exhibit disease-specific transcriptional fates

**Fig. 3:**
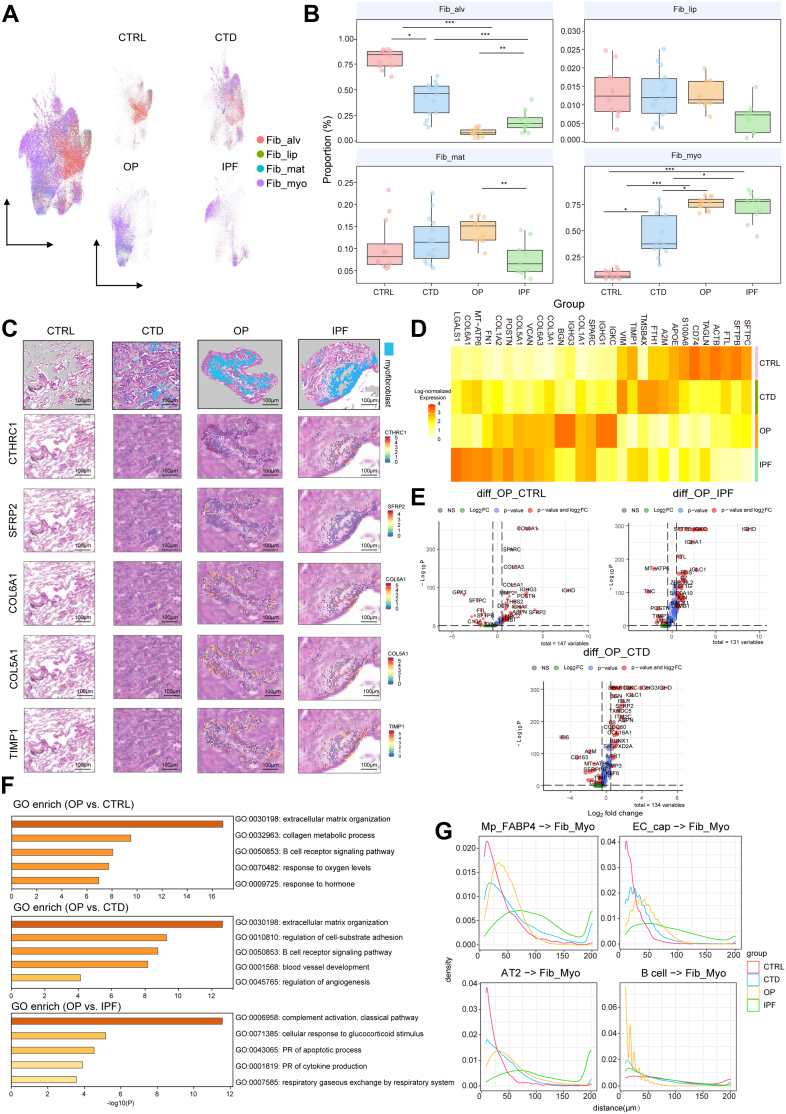
Myofibroblast heterogeneity and spatial niches in reversible versus progressive lung fibrosis. A) Uniform manifold approximation and projection (UMAP) plot showing the distribution of mesenchymal cell types in lung tissues by spatial transcriptomic (ST) profiling. B) Boxplots demonstrating cell proportions of alveolar fibroblast, lipofibroblast, matrix fibroblast and myofibroblast across groups from ST profiling. Group comparisons by Kruskal–Wallis with Benjamini–Hochberg correction; median and interquartile range (IQR; [Q3−Q1]); ∗*P* < 0.05; ∗∗*P* < 0.01; ∗∗∗*P* < 0.001; n = 5 (CTRL, CTD-ILD, IPF); n = 6 (OP). C) Spatial expression of fibrosis-related genes in Fib_myo overlaid on pathology images. Scale bar, 100 μm. D) Heatmap of differentially expressed genes (DEGs) in Fib_myo spots across four groups from ST profiling data. Scale = log-normalised expression. E–F) DEGs by volcano plot (E) and Gene Ontology (GO) terms (F) for OP Fib_myo vs. controls (CTRL), connective tissue disease-associated interstitial lung disease (CTD-ILD), and idiopathic pulmonary fibrosis (IPF). Bar plots showing GO terms from upregulated genes in OP. The representative genes are labelled. Dashed lines demarcate two-sided Benjamini-Hochberg-corrected *P* adj = 0.05 and log 2 fold change (FC) = 0.25. G) Distance density distributions between Fib_myo spots and key celltype-spots in alveoli across four groups from ST profiling data.

**Supplementary Figure S3:**
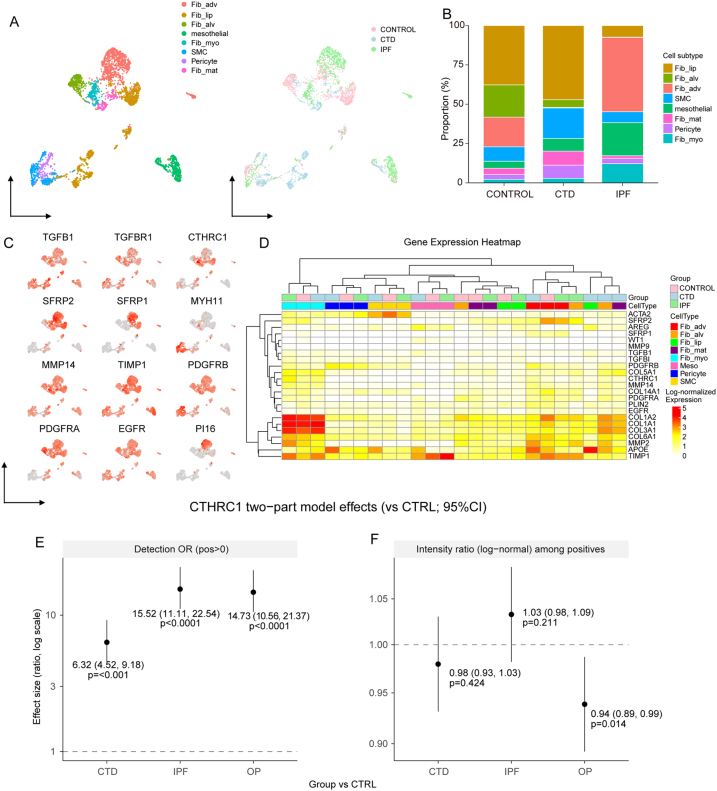
Single-cell RNA sequencing (scRNA-seq) and spatial transcriptomic (ST) landscapes reveal distinct gene expression signatures of fibroblast subpopulations. A) Uniform manifold approximation and projection (UMAP) plots of eight types of fibroblasts in lung tissues of controls (CTRL), patients with connective tissue disease-associated interstitial lung disease (CTD-ILD) and idiopathic pulmonary fibrosis (IPF). B) Fractions of fibroblast subtypes in scRNA-seq from CTRL, CTD-ILD, and IPF groups. C) Selected repair and fibrosis gene markers are visualised in the UMAP space across all fibroblasts. D) Scaled expression levels of repair and fibrosis genes with altered expression across fibroblast types in the three groups. Scale = log-normalised expression. E) Forest plot of odds ratios (OR) and 95% CI for CTHRC1 detection (expr >0) by group relative to CTRL: CTD OR ≈ 6.32 [4.52, 9.18], OP OR ≈ 14.73 [10.56, 21.37], IPF OR ≈ 15.52 [11.11, 22.54]; all *P* < 0.001. Dashed line: OR = 1 (null); x-axis on log scale; n = 5 (CTRL, CTD-ILD, IPF groups); n = 6 (OP group). F) Forest plot of fold-change ratios and 95% CI for per-positive CTHRC1 intensity (log-normal model, exponentiated coefficients) versus CTRL: OP ≈ 0.94 (95% CI 0.89–0.99, *P* = 0.014), CTD ≈ 0.98 (95% CI 0.93–1.03, *P* = 0.424), IPF ≈ 1.03 (95% CI 0.98–1.09, *P* = 0.211). Dashed line: ratio = 1 (null, log scale); n = 5 (CTRL, CTD-ILD, IPF groups); n = 6 (OP group).

Integrated scRNA-seq and spatial transcriptomics identified a progressive decrease in alveolar fibroblasts (Fib_alv) during fibrotic progression in CTD-ILD and IPF lungs (Supplementary Figure S3A and B). Supplementary Figure S3C illustrates the projection of functional genes onto the single-cell UMAP of lung fibroblasts. Clustering analysis demonstrated separable identities for myofibroblasts (Fib_myo), adventitial fibroblasts (Fib_adv), pericytes, and smooth muscle cells, while Fib_alv showed partial overlap with matrix-producing fibroblasts (Fib_mat) and inflammatory lipofibroblast (Fib_lip), suggesting functional similarities among these cells in the lung (Supplementary Figure S3D). Dimensionality reduction and stromal cell spot clustering of ST data performed after deconvolution of scRNA-seq data revealed distinct distribution patterns of stromal subpopulations (Fig. 3A). The proportions of Fib_myo spots were significantly increased in OP and IPF, with the significant decrease of Fib_alv, whereas CTD-ILD exhibited transitional characteristics between Fib_alv spots and Fib_myo spots (Fig. 3B). Notably, lung Fib_myo spots in OP, CTD-ILD, and IPF exhibited substantially higher CTHRC1 detection than CTRL (Fig. 3C), a marker of fibroblast activation, while per-positive intensity is largely unchanged except for a modest decrease in OP (Supplementary Figure S3E and F). Differentially expressed genes (DEGs) analysis indicated that OP Fib_myo spots uniquely showed B-cell-related immunoglobulin genes (IGHs), while IPF Fib_myo spots exhibited fibrotic remodelling markers (POSTN, FN1). OP-derived Fib_myo spots exhibited similarities to those in IPF in terms of collagen expression, metabolic activity, and lung repair-related pathways, but displayed opposing features compared with CTD-ILD-associated Fib_myo spots (Fig. 3D and E). GO functional pathway enrichment showed that OP Fib_myo spots significantly up-regulated the BCR pathways compared with the CTRL and CTD-ILD groups, and the GC responsiveness and apoptosis pathways compared with the IPF group (Fig. 3F). Spatial proximity analyses further confirmed that Fib_myo spots preferentially co-localised with alveolar B cell-spots in OP, whereas their distances to FABP4^+^ macrophage, AT2, and endothelial cell-spots were increased compared with CTRLs (Fig. 3G).

### Progenitor-like features in alveolar fibroblasts associated with myofibroblast fate

**Supplementary Figure S4:**
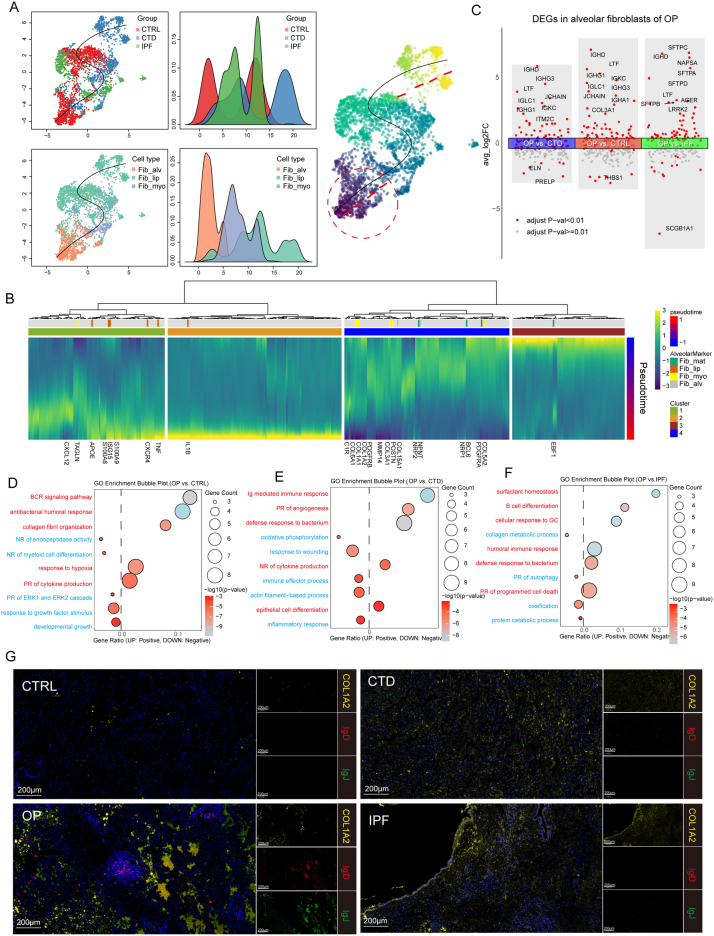
Gene expression patterns from alveolar fibroblasts to myofibroblast. A) UMAP of group and fibroblast subtypes (left) with pseudotime trajectory analysis (right). Ridgeline plots quantify trajectory distributions. B) Slingshot pseudo-time ordering of fibroblasts and heatmap showing normalised expression of selected genes along pseudo-time using rolling average expression in single-cell RNA sequencing (scRNA-seq) data. C) Volcano plots of differentially expressed genes (DEGs) in Fib_alv spots (OP vs. controls (CTRL), connective tissue disease-associated interstitial lung disease (CTD-ILD), and idiopathic pulmonary fibrosis (IPF). D-F) Gene Ontology (GO) term enrichment for Fib_alv DEGs in OP vs. CTRL (D), CTD-ILD (E), and IPF (F) in spatial transcriptomic (ST) profiling data (Benjamini-Hochberg [BH]-adjusted q < 0.05). G) Spatial locations of fibroblasts (marked by COL1A2) and B-cells (marked by IgD and IgJ) in the groups of CTRL, early phases of OP, CTD-ILD, and IPF. Scale bar, 200 μm (magnified image).

**Supplementary Figure S5:**
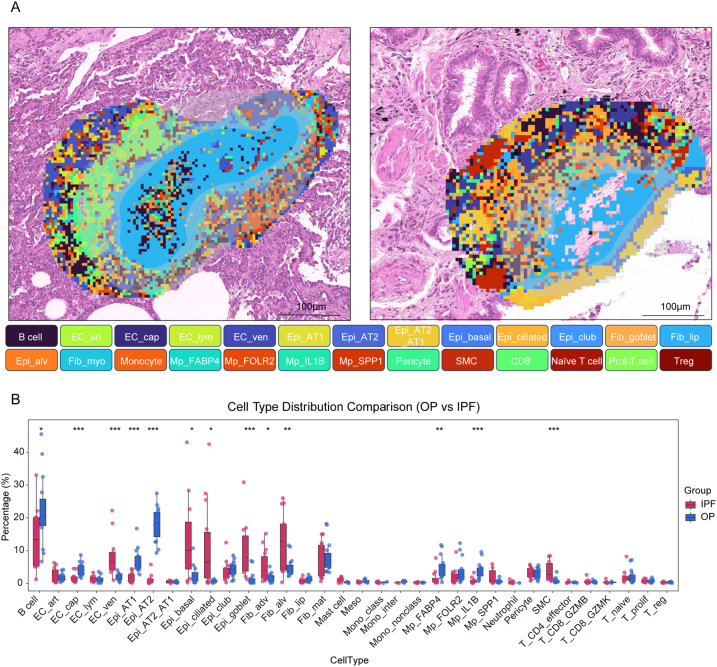
Cell composition around typical lesions in organising pneumonia (OP) and idiopathic pulmonary fibrosis (IPF). A) Delimitation of cells around lesions in OP and IPF. Scale bar, 100 μm (magnified image). B) Comparison of differences in proportions of various cell subtypes around lesions in OP and IPF. Group comparisons by Wilcoxon rank-sum test; median and interquartile range (IQR; [Q3−Q1]); ∗*P* < 0.05; ∗∗*P* < 0.01; ∗∗∗*P* < 0.001; n = 3 ROIs per sample; n = 5 (IPF group); n = 6 (OP group).

Pseudo-time trajectory analysis identified Fib_alv as progenitor cells (enriched in CTRL), differentiating toward Fib_myo, with Fib_lip as terminal cells in CTD-ILD^20^^,^^21^ (Supplementary Figure S4A). Transcriptomic shifts revealed functional transitions. Early Fib_alv expressed cell differentiation, ECM and apoptosis regulators (EBF1, PDGFRA, BCL6); intermediate Fib_myo upregulated collagen metabolism (COL1A1, COL1A2, COL6A1) and repair genes (POSTN, MMP14, NPNT); later stages showed reduced collagen genes but increased inflammation markers (IL1B, TNF, S100A9) (Supplementary Figure S4B). OP Fib_alv spots showed unique upregulation of IGHD, LTF, and surfactant genes (SFTPD, NAPSA) (Supplementary Figure S4C). Comparative pathway analysis indicated OP-specific enrichment in antibacterial responses, B-cell differentiation, and GC-mediated apoptosis, distinct from the inflammatory/oxidative stress and ECM remodelling signatures (Supplementary Figure S4D–F). The spatial relationship between IgD + B cells and secreted IgJ, as well as COL1A2+ fibroblasts, was confirmed by immunofluorescence (Supplementary Figure S4G). Peri-lesion mapping further distinguished the fibrotic lesions in OP from those in IPF (Supplementary Figure S5A and B).

### Glucocorticoid response and apoptosis define the reversible microenvironment

**Fig. 4:**
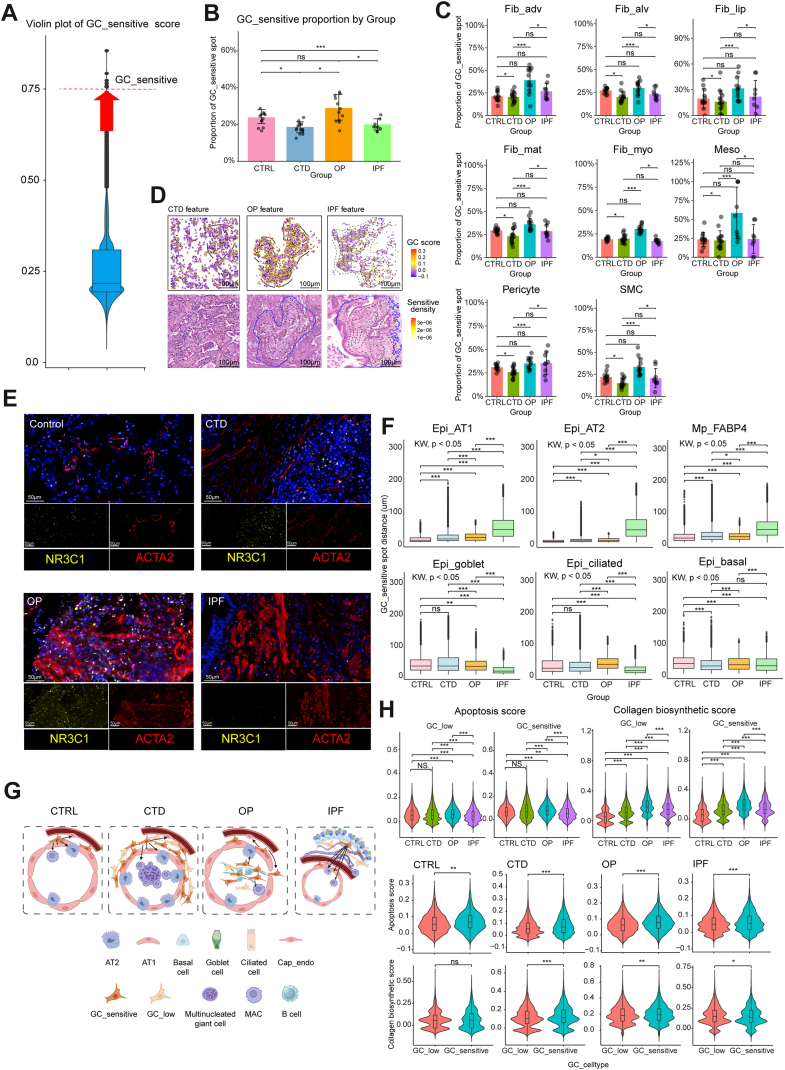
Spatial characterisation of a glucocorticoid (GC)–sensitive myofibroblast state linked to a pro-resolution niche in reversible fibrosis. A) Schematic of GC-sensitive spot selection criteria. Cells with GC_Response scores in the top 25% are considered GC-sensitive spots. B) GC-sensitive spot (HD Visium 8 μm pixel unit carrying spatial transcriptomic measurements) proportion by group at the donor level. For each donor, the GC-sensitive spot proportion was computed as the ROI-weighted percentage of GC-sensitive fibroblast spots among all fibroblast spots; each dot denotes one donor. Group comparisons by Kruskal–Wallis with Benjamini–Hochberg correction; mean ± SEM; ∗*P* < 0.05; ∗∗∗*P* < 0.001; n = 5 (CTRL, CTD-ILD, IPF); n = 6 (OP). C) GC-sensitive proportion by group at the donor level. For each donor, the GC-sensitive proportion was computed as the ROI-weighted percentage of GC-sensitive fibroblast spots among all fibroblast spots; each dot denotes one donor. Group comparisons by Kruskal–Wallis and pairwise contrasts by Dunn's test with Holm adjustment; mean ± SEM; ∗*P* < 0.05; ∗∗∗*P* < 0.001; n = 5 (CTRL, CTD-ILD, IPF); n = 6 (OP). D) The spatial contour map overlaid with a density map illustrates the distribution characteristics of GC-sensitive spots. E) Immunofluorescence (IF) validation of glucocorticoid receptor (NR3C1) with ACTA2+ Fib_myo in CTRL, CTD-ILD, OP and IPF. Scale bar, 50 μm (magnified image). F) Box plots showing the spatial proximity of GC-sensitive Fib_myo spots with FABP4+ macrophage and alveolar epithelium spots in OP group, and with bronchial epithelium in IPF group. Pairwise comparisons of intercellular distances across groups were conducted using the nonparametric Wilcoxon rank-sum test; median and interquartile range (IQR; [Q3−Q1]); ∗*P* < 0.05; ∗∗∗*P* < 0.001; n = 5 (CTRL, CTD-ILD, IPF groups); n = 6 (OP group). G) Schematic depicting the cellular microenvironment of GC-sensitive myofibroblasts, highlighting their spatial relationships with alveolar macrophages, endothelium, and epithelium across CTRL, OP, CTD-ILD, and IPF. H) Violin plot showing the gene set scores of apoptosis and collagen biosynthesis in GC-sensitive vs. GC-low fibroblasts across different groups. Group comparisons by Kruskal–Wallis test with Dunn's post hoc test; mean ± SEM; ∗*P* < 0.05; ∗∗*P* < 0.01; ∗∗∗*P* < 0.001; n = 5 (CTRL, CTD-ILD, IPF); n = 6 (OP).

**Supplementary Figure S6:**
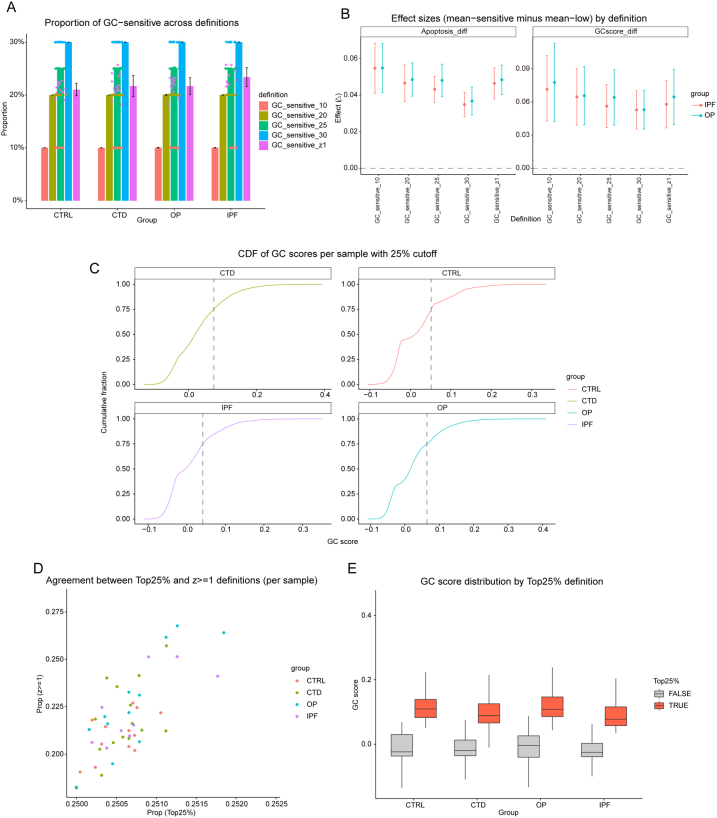
Robustness and validation of GC-sensitive fibroblast spots definitions across multiple thresholding strategies. A) Proportion of GC-sensitive fibroblast spots by group under multiple definitions (Top 10/20/25/30 and z-threshold). B) Tabulated effect sizes and 95% CIs for GC_Response_score and Apoptosis score in OP and IPF across definitions, supporting threshold-agnostic conclusions. C) Per-sample cumulative distribution functions (CDFs) of GC_Response_score with vertical lines marking the Top 25% cutoff, illustrating within-sample thresholding on the native score distribution. D) Per-sample scatter of GC-sensitive proportions under Top25% (x-axis) versus z-threshold (y-axis) with the y = x reference, indicating z-threshold selects fewer cells while preserving overall concordance. E) GC_Response_score distributions by group with cells stratified by the Top25% definition, showing higher GC_Response_score among GC-sensitive cells across groups; median and interquartile range (IQR; [Q3−Q1]).

To delineate glucocorticoid (GC)-sensitive microenvironments, we scored fibroblast spots using the ‘CELLULAR_RESPONSE_TO_GLUCOCORTICOID_STIMULUS’ gene set from MSigDB via AddModuleScore_UCell. A sensitivity analysis confirmed that the positive and significant effect of the resulting GC_Response score in OP and IPF was robust across a range of thresholds (Top 10–Top30% percentiles and a z-score, Supplementary Figure S6A–C). This was further supported by consistent Spearman correlations and bootstrap confidence intervals from 1000 replicates, which lay well above zero (Supplementary Figure S6D and E). Validating the Top 25% threshold as clear and robust, the top quartile of spots with elevated GC_Response scores were thus classified as the GC-sensitive subset (Fig. 4A). OP lungs exhibited a higher GC-sensitive spots proportion among fibroblasts than IPF (Fig. 4B). Across stromal subtypes, OP donors showed the highest fraction of GC-sensitive Fib_myo spots (Fig. 4C). Kernel density maps revealed dense, diffuse GC-sensitive clusters in OP versus sparse distributions in IPF, in line with NR3C1 staining (Fig. 4D and E). In OP, GC-sensitive Fib_myo spots were similarly close to alveolar epithelium and alveolar macrophages; in IPF, it shifted away from the alveolar niche toward the bronchial niche (Fig. 4F and G). Although OP lung fibroblast spots exhibited the highest collagen biosynthetic score, their apoptotic scores remained the highest among four groups. IPF lung fibroblast spots displayed the lowest apoptotic priming score (Fig. 4H).

### A pro-apoptotic, glucocorticoid-sensitive myofibroblast state distinguishes reversible OP from progressive IPF

**Fig. 5:**
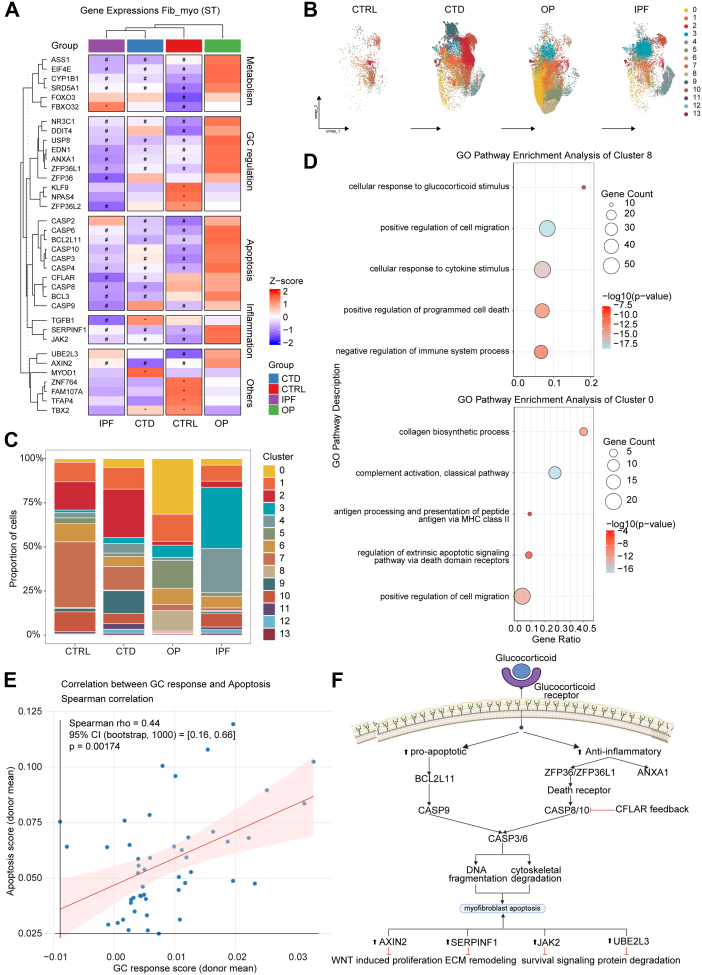
Myofibroblast heterogeneity uncovers a pro-apoptotic subtype mediating GC response in reversible fibrosis. A) Donor-level heatmap of GC-Response and related pathway genes in Fib_myo spots (HD Visium, 8 μm bins). Values are donor means (Z-score). Group comparisons use Kruskal–Wallis followed by Dunn's test with Benjamini-Hochberg correction; symbols indicate significant contrasts versus OP (∗ higher than OP; # lower than OP; *P* < 0.05; n = 5 [CTRL, CTD-ILD and IPF groups]; n = 6 [OP group]). B) UMAP of Fib_myo spots clustered by shared nearest neighbour (SNN) graph. Small clusters (n < 300) were flagged as reference. C) Proportions of 14 clusters in each myofibroblast cluster according to disease state. D) GO enrichment of upregulated DEGs of cluster 0 and 8 using BH-adjusted q < 0.05. E) Donor-level Spearman correlation between GC response and apoptosis scores in Fib_myo. 95% CI (bootstrap, n = 1000), and *P* values shown; line indicates least-squares fit for visualisation. F) Proposed GC-mediated apoptotic mechanism in OP Fib_myo.

**Supplementary Figure S7:**
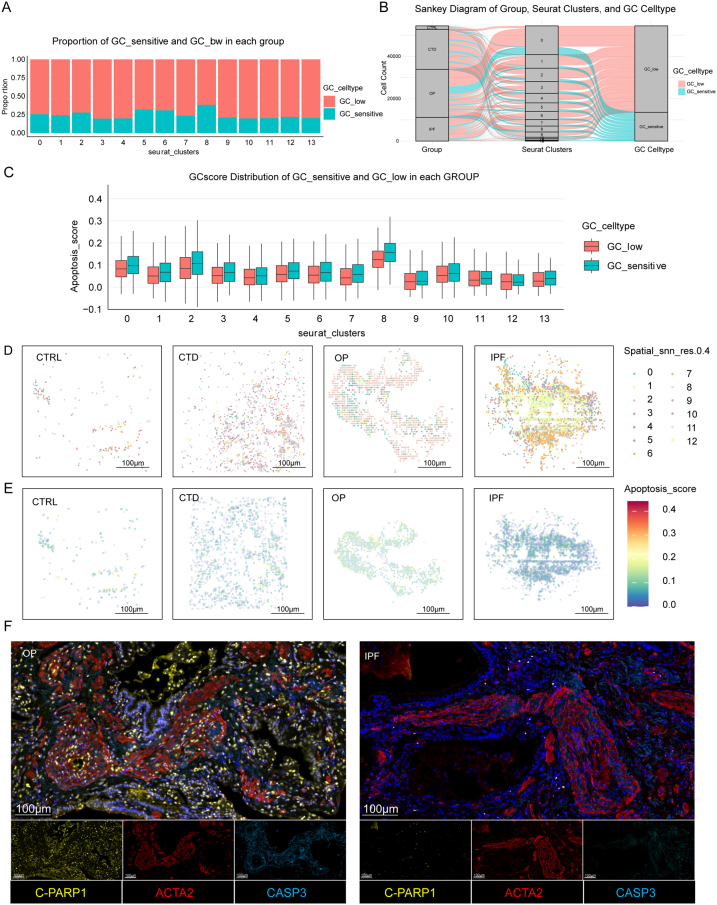
Spatial distribution of glucocorticoid (GC)-sensitive fibroblasts. A) Proportions of GC-sensitive and GC-low cell types in each myofibroblast cluster according to disease state. B) Sankey diagram illustrating the relationship between GC-sensitive cell subpopulations, disease states, and myofibroblast clusters. C) Comparison of apoptosis scores in GC-sensitive and GC-low cell spots in various clusters of myofibroblasts; mean ± SEM. D) Cluster map of myofibroblasts in representative regions of interest (ROIs). Scale bar, 100 μm (magnified image). E) Apoptosis scores in myofibroblasts in representative ROIs. Scale bar, 100 μm (magnified image). F) IF validation of apoptosis markers (cleaved PARP1 and CASP3) co-localised with ACTA2+ Fib_myo in OP and IPF. Scale bar, 100 μm.

Spatial transcriptomic profiling identified Fib_myo spots with distinct GC responsiveness in ILDs. Comparative analysis revealed pronounced upregulation of GC signalling (NR3C1), apoptosis effectors (BCL2L11, CASP3/6/8/9/10), and stress regulators (DDIT4, ZFP36L1) in OP-derived lung Fib_myo spots, along with the key signalling regulators AXIN2, JAK2, and SERPINF1 (Fig. 5A). Notably, the caspase protease family demonstrated particularly significant activation patterns, with multiple isoforms showing coordinated overexpression. Unsupervised clustering partitioned Fib_myo spots into 14 subsets with disease-specific enrichment patterns (Fig. 5B). CTRL lungs predominantly harboured cluster 7, while CTD-ILD lungs showed clusters 2. Remarkably, OP-specific Fib_myo spots clusters 0 and 8 exhibited preferential activation of GC response pathways and apoptosis regulation (Fig. 5C and D), with cluster 8 demonstrating the maximal GC-sensitive Fib_myo proportion (Supplementary Figure S7A and B). IPF Fib_myo spots lacked analogous GC-responsive subsets (Sankey diagram, Supplementary Figure S7B). Apoptosis scoring indicated that cluster 8 had higher levels of apoptosis (Supplementary Figure S7C), resulting in increased apoptosis scores in OP (Supplementary Figure S7D and E). OP myofibroblasts (ACTA2+) exhibited significantly higher co-expression of both CASP3 and c-PARP1 compared to those in IPF tissues (Supplementary Figure S7F). Correlation analysis of GC response and apoptosis scores demonstrated a significant positive correlation between the two metrics (Fig. 5E). These data are consistent with the possibility that OP reversibility may arise from spatially restricted, GC-primed myofibroblast niches capable of apoptosis-driven resolution—a pattern not observed in our IPF samples. The tight coupling of GC response and apoptotic activation (Fig. 5F) provide a mechanistic basis for the therapeutic efficacy of corticosteroids in OP, in contrast to their ineffectiveness in IPF.

### The OP lesion follows a programmed trajectory from inflammation and apoptosis to final resolution

**Fig. 6:**
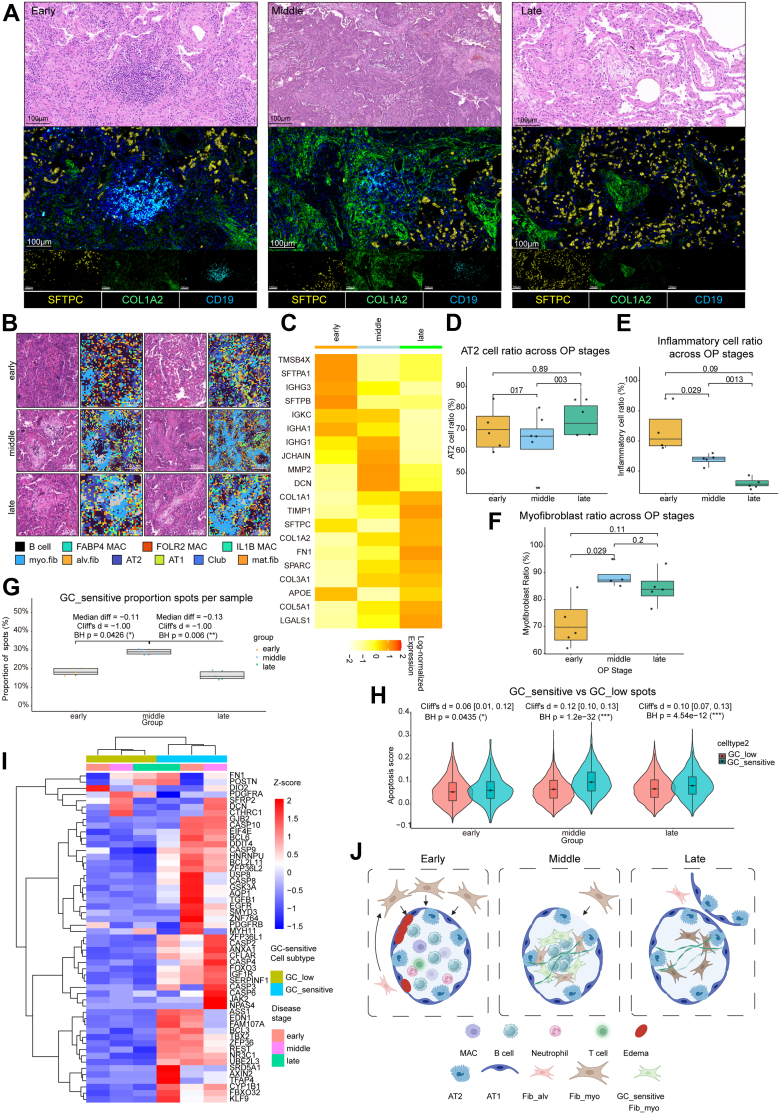
Spatiotemporal evolution of glucocorticoid (GC)-sensitive Fib_myo in organising pneumonia (OP). A) Paired H&E and corresponding multiplex immunofluorescence (IF) images illustrating the spatial distribution of fibroblasts (COL1A2, green), alveolar type 2 cells (SFTPC, yellow), and B cells (CD19, cyan) across the Early, Middle, and Late phases of OP. Top panels: H&E delineate alveolar architecture and intraluminal buds/plugs characteristic of each phase. Bottom panels: Paired IF validates phase-specific localisation patterns of fibroblasts, AT2 cells, and B cells within the same regions. Scale bar, 100 μm (magnified image). B) Stage-specific cellular distributions (early/middle/late OP) in spatial transcriptomic (ST) profiling data. Two fields are selected for each stage, with pathological images on the left side of each field and cell distributions defined by the corresponding ST data on the right. C) Heatmap showing the OP stage-specific differentially expressed genes (DEGs) from ST data. Scale = log-normalised expression. D–F) Dynamic changes in proportions of AT2 spots (D), inflammatory cell spots (macrophages, lymphocytes, neutrophils; E), Fib_myo spots (F) from early to late phases of OP that statistics are at the donor level. Group comparisons by Kruskal–Wallis with Benjamini-Hochberg correction; median and interquartile range (IQR; [Q3−Q1]); ∗*P* < 0.05; ∗∗*P* < 0.01; n = 6 (OP group). G) Sample-level definition of GC-sensitive Fib_myo as spots at or above the within-sample 75th percentile of GC-Response score. Proportions differ by stage, with middle OP significantly higher than early and late (Kruskal–Wallis with BH-adjusted Dunn tests; effect sizes: Cliff's delta); median and interquartile range (IQR; [Q3−Q1]); ∗*P* < 0.05; ∗∗*P* < 0.01; n = 6 [OP group]). H) Within Fib_myo spots, GC_sensitive spots show higher apoptosis scores than GC_low overall and within stages (Wilcoxon tests with BH adjustment; effect sizes: Cliff's delta); mean ± SEM; ∗*P* < 0.05; ∗∗∗*P* < 0.001; n = 6 (OP group). I) Heatmap of repair/fibrosis/GC pathway genes in Fib_myo spots during disease progression. Scale = gene-wise Z-score. J) Schematic of Fib_myo-B-cell niche co-evolution. In the initial stages of OP, the alveolar cavity exhibits a dense population of B-cells, concomitant with damage to the alveolar epithelium. Subsequently, myofibroblasts migrate into the alveolar space, presumably driven by chemotactic signals from B-cells, and demonstrate heightened sensitivity to GCs during this phase. In the later stages of OP, B-cells progressively diminish, while senescent myofibroblasts exhibit reduced sensitivity to GCs, coinciding with decreased apoptotic activity.

Based on the well-recognised fibrotic progression and the distribution of immune cells and fibroblasts in OP alveoli, we delineated three histologically and IF-assisted distinct phases of OP progression (Fig. 6A). Early lesions showed diffuse CD19^+^ B-cell infiltration and alveolar serous exudation, with peripheral fib_myo clusters adjacent to inflammatory foci (Fig. 6B). Middle-stage lesions featured fibroblast migration into alveolar spaces forming immune-encapsulated “soft-core” structures, while late-stage lesions developed collagen-enriched plugs with diminished inflammation yet preserved alveolar architecture (Fig. 6A and B). In the early stage, B-cell activity markers (IGKC, IGHG3, IGHA1) dominated, the middle stage transitions to fibroblast-driven ECM remodelling and plasma cell maturation (DCN, MMP2, JCHAIN), and the late stage features upregulation of alveolar epithelial repair genes but fibrotic persistence, indicating pharmacologic intervention is indispensable (TIMP1, SFTPC) (Fig. 6C). Cellular niche quantification demonstrated progressive inflammatory cell ratio (composed of macrophages, lymphocytes and neutrophils spots) reduction with dynamic myofibroblast redistribution, accompanied by late-phase restoration of AT2 epithelial cell-spots (Fig. 6D–F).

Fibrotic foci exhibited stage-specific molecular reprogramming, with Fib_myo apoptosis scores peaking during intermediate phase while GC sensitive spot proportions declined in late lesions (Fig. 6G and H). Transcriptomic analyses revealed functional spatial partitioning. GC-sensitive regions were enriched in apoptotic executers, especially in middle phase (CASP2/3/4/6/9, BCL2L11), whereas GC-low areas progressively upregulated fibrotic mediators (FN1, POSTN, DIO2, CTHRC1) (Fig. 6I), indicating fibrotic/repair genes gradually increased in GC-low regions during progression, particularly in the late phase, whereas apoptotic gene expression in GC-sensitive zones peaked in the mid-stage before declining, reflecting a dynamic equilibrium between cell death programs and reparative remodelling (Fig. 6J).

### B cell–derived MIF promotes migration of GC-responsive fibroblasts via CD74

**Fig. 7:**
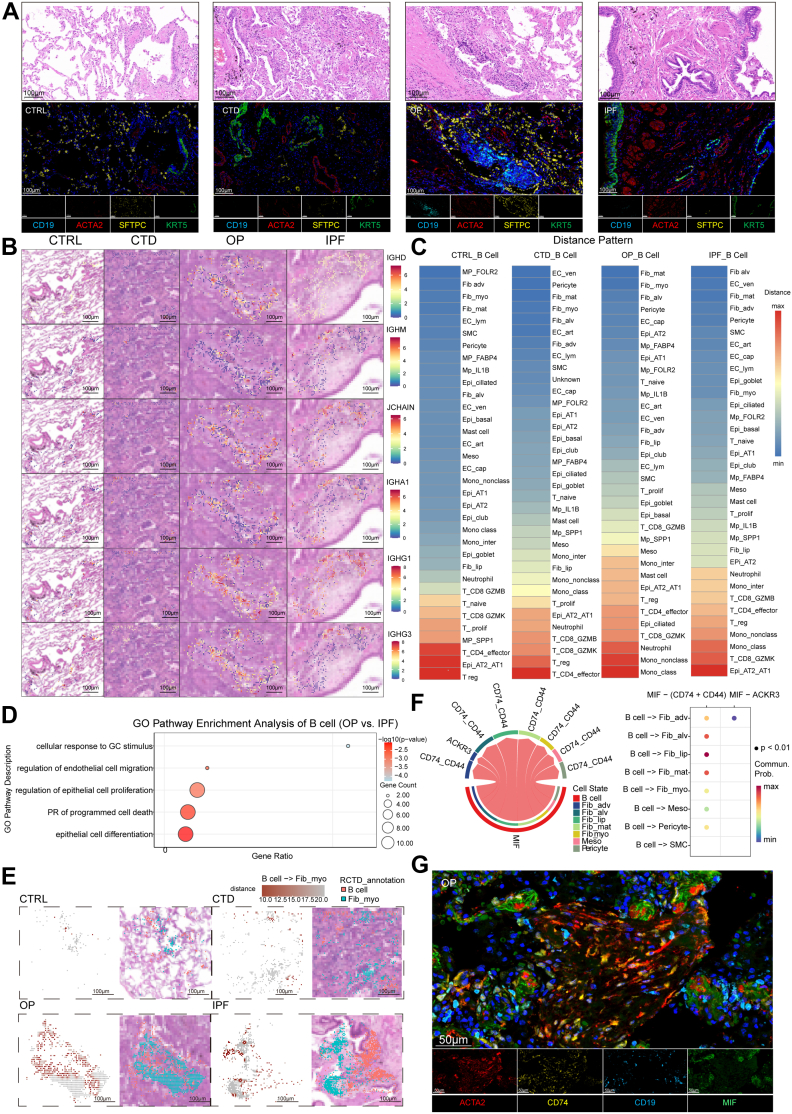
A distinct B-cell population shapes a pro-resolution myofibroblast niche in reversible fibrosis. A) Paired H&E and corresponding multiplex immunofluorescence (IF) images across groups (CTRL, CTD-ILD, OP, IPF). Top row: H&E staining delineates alveolar architecture and intraluminal plugs/fibroinflammatory buds at the corresponding regions. Bottom row: multiplex IF showing CD19 (B cells, cyan), ACTA2 (myofibroblasts, red), SFTPC (AT2 cells, yellow), and KRT5 (airway basal cells, green). Scale bar, 100 μm (IF panels). B) Spatial expression of B-cell markers across four groups, with the pathological images of each group as the background. Scale bar, 100 μm. C) The gradient colour scale shows the ordering of distance from B-cells in each group. D) Gene Ontology (GO) term enrichment from upregulated genes in B-cells by OP to IPF comparison (BH-adjusted q < 0.05). E) Comparison of spatial distance between B-cells and fib_myo among the four groups. Scale bar, 100 μm. F) The prediction of ligand-receptor interactions activity from B-cell to fibroblasts through “Cellchat” algorithm from single-cell RNA sequencing (scRNA-seq) data. G) IF showing ligand (MIF)–receptor (CD74) co-localisation in CD19+ B cells and ACTA2+ Fib_myo within the same field in OP. Scale bar, 50 μm.

**Supplementary Figure S8:**
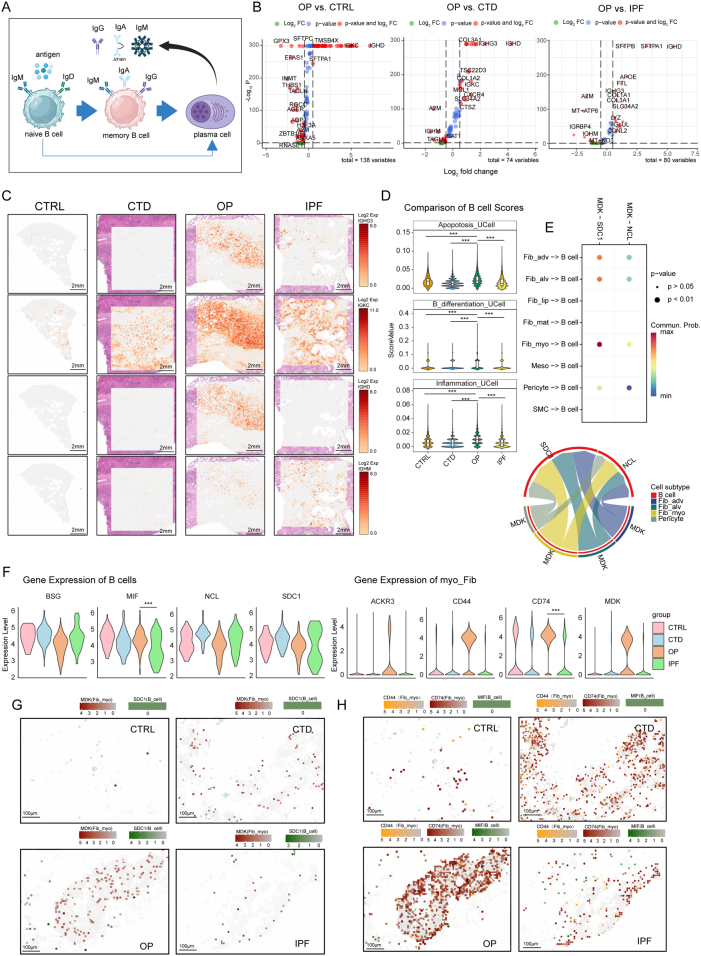
Myofibroblast and B-cell interactions in fibrotic interstitial lung disease (fILD). A) Schematic of B-cell differentiation. B) Volcano plot showing the differentially expressed genes (DEGs) in B-cells in the OP group compared with controls (CTRL), connective tissue disease-associated interstitial lung disease (CTD-ILD), and idiopathic pulmonary fibrosis (IPF). The representative genes are labelled. Dashed lines: BH-adjusted *P* adj = 0.05 and log 2 FC = 0.25. C) Expression of IGHG3, IGKC, IGHD, and IGHM in the entire spatial transcriptomic (ST) landscape of the four groups. Scale bar, 2 mm. D) Violin plots showed apoptosis, B-cell differentiation, and inflammation scores in B-cell spots among four groups. Pairwise group comparisons of module scores were performed using Wilcoxon rank-sum tests with BH adjustment, after assessing overall differences with Kruskal–Wallis tests; mean ± SEM; ∗∗∗*P* < 0.001; n = 5 (CTRL, CTD-ILD, IPF groups); n = 6 (OP group). E) Fibroblasts interacting with B-cells through several MDK ligand-receptor pairs in single-cell RNA sequencing (scRNA-seq). F) Expression of ligand-receptor pairs (BSG, MIF, SDC1, and NCL in B-cells, and CD44, CD74, MDK, and ACKR3 in Fib_myo) in the ST dataset. Pairwise group comparisons were performed using Wilcoxon rank-sum test with Benjamini-Hochberg adjustment; mean ± SEM; ∗∗∗*P* < 0.001; n = 5 (CTRL, CTD-ILD, IPF groups); n = 6 (OP group). G-H) Co-localisation of MDK (G) and MIF (H) ligand-receptor pair-expressing myofibroblasts and B-cells in ST profiling. Scale bar, 100 μm.

Existing literature suggests B-cell involvement in fILD pathophysiology.^22^^,^^23^ IF staining of lung sections for typical markers suggested the spatial proximity of myofibroblasts, AT2 and B-cells in OP (Fig. 7A, Supplementary Figure S4G). Peri-lesion mapping further distinguished fibrotic lesions in OP from those in IPF (Supplementary Figure S5A and B), suggesting distinct regulatory mechanisms underlying fibrosis development. Spatial transcriptomic profiling revealed a pathogenic B-cell-Fib_myo axis unique to OP, where fibrotic lesions exhibited significantly elevated IGHD/IGHG3 expression compared with IGHM-dominant patterns in IPF (Fig. 7B, Supplementary Figure S8A–C). Spatial mapping confirmed dense IGH enrichment within alveolar-proximal B-cell niches—contrasting sharply with vascular B-cell-spots localisation in comparator diseases—with critical co-localisation to Fib_myo spots, AT2, and capillary spots establishing OP-specific microanatomical relationships (Fig. 7C). Functional assessment further suggested OP B-cell-spots specialisation through GO-enriched epithelial proliferation modulation, apoptotic regulation, and glucocorticoid response pathways (Fig. 7D), corroborated by elevated B-cell differentiation/apoptosis and B-cell inflammation scores (Supplementary Figure S8D). Specifically, the concurrent aggregation of B cells and myofibroblasts within the alveolar space resulted in significantly reduced intercellular distances in OP compared with other groups (Fig. 7E). Receptor-ligand analysis using integrated scRNA and spatial data elucidated bidirectional crosstalk mechanisms between B-cells and myofibroblasts in the lung. B-cell-derived MIF recruited myofibroblasts via CD74^+^/CD44^+^ receptors, while fib_myo-secreted MDK engaged SDC1^+^ B-cells to regulate immune functions (Fig. 7F, Supplementary Figure S8E–H), collectively forming an OP-exclusive regulatory circuit, a finding validated by IF staining in OP tissue (Fig. 7G). These spatially resolved interactions distinguished OP from IPF, where minimal B-cell-fib_myo proximity was observed.

**Supplementary Figure S9:**
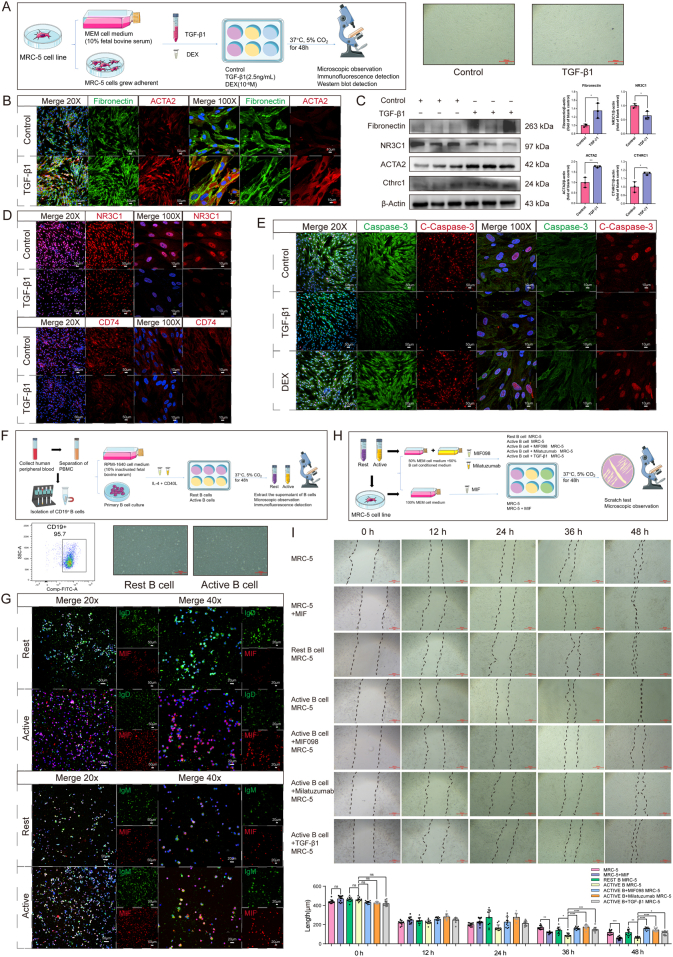
B cell–derived MIF–CD74 drives migration of GC-sensitive fibroblasts. (A) Human lung fibroblasts (MRC-5) were stimulated with TGF-β1 to induce a myofibroblast-like phenotype. (B)Immunofluorescence images showing α-smooth muscle actin (ACTA2, red) and fibronectin (green) expression in control and TGF-β1–treated MRC-5 cells; TGF-β1 stimulation markedly increased ACTA2 and fibronectin expression. Scale bars, 50 μm and 10 μm (magnified image). (C) Western blot analysis of the fibroblast/myofibroblast markers fibronectin, CTHRC1 and ACTA2, MIF receptor CD74, together with the glucocorticoid receptor NR3C1, in control and TGF-β1–treated MRC-5 cells. Group comparisons were performed using unpaired Student's t-test; mean ± SEM; ∗∗∗*P* < 0.001; n = 3 replicates. (D) Immunofluorescence staining of NR3C1 showing ligand-induced nuclear translocation in control MRC-5 cells and reduced NR3C1 expression with attenuated nuclear accumulation after TGF-β1 stimulation. CD74 was highly expressed in control MRC-5 cells but decreased after TGF-β1 intervention. Scale bars, 50 μm and 10 μm (magnified image). (E) Immunofluorescence detection of the apoptosis markers caspase-3 and cleaved caspase-3 (C-caspase-3) and their co-localisation in MRC-5 cells; TGF-β1 treatment decreased caspase-3/cleaved caspase-3 signals. In contrast, DEX-treated MRC-5 cells exhibited increased caspase-3 and cleaved caspase-3 staining. Scale bars, 50 μm and 10 μm (magnified image). (F) Human peripheral blood B cells were isolated and separated into resting B cells (Rest) and *invitro*–activated B cells (Active). (G) Immunofluorescence analysis of MIF secretion (red) and B cell surface immunoglobulins IgD (green) and IgM (green); Rest B cells predominantly expressed IgD, whereas Active B cells exhibited increased MIF secretion and elevated IgM expression. Scale bars, 50 μm and 20 μm (magnified image). (H) Conditioned media from resting or activated B cells, or a B cell–MRC-5 co-culture system, were used to simulate the *in vivo* microenvironment. Activated B cells were treated with the MIF inhibitor MIF098, and MRC-5 cells were treated with the CD74-blocking antibody Milatuzumab to inhibit MIF–CD74 signalling. (I) Scratch-wound migration assay of MRC-5 cells under the indicated conditions. Exogenous MIF protein or co-culture with activated B cells enhanced MRC-5 migration, whereas inhibition of MIF with MIF098 or blockade of CD74 with Milatuzumab reduced migration. In contrast, when activated B cells were co-cultured with TGF-β1–pretreated (myofibroblast-like) MRC-5 cells, migration was not further promoted. Statistical significance for selected pairwise comparisons is indicated in the figure. Data are presented as mean ± SEM; n = 3 replicates. *∗P* < 0.05; *∗∗P* < 0.01; *∗∗∗P* < 0.001 (one-way ANOVA with Tukey's multiple comparisons test). Scale bar, 200 μm.

To mechanistically probe this axis, we performed targeted perturbation in human lung fibroblasts. We chose MRC-5 as a glucocorticoid-sensitive, homoeostatic human lung fibroblast model that reliably exhibits baseline fibroblast features and reproducible GC responses, and used TGF-β–treated MRC-5 to emulate the disease–relevant transition to a myofibroblast-like, glucocorticoid-refractory state.^24^, ^25^, ^26^, ^27^, ^28^, ^29^ TGF-β1 stimulation increased ACTA2, fibronectin and CTHRC1, reduced CD74 expression and NR3C1 expression/nuclear translocation, and attenuated caspase-3/cleaved caspase-3 staining in MRC-5. DEX-treated MRC-5 cells exhibited increased caspase-3 and cleaved caspase-3 staining, suggesting enhanced apoptotic activity (Supplementary Figure S9A–E). Isolated human peripheral blood B cells were separated into resting and activated subsets; activated IgD + B cells showed higher MIF secretion and an IgD + -to-IgM + switch (Supplementary Figure S9F and G). In a co-culture system, conditioned media or direct contact from activated B cells, as well as exogenous MIF, significantly accelerated scratch-wound migration of GC-responsive MRC-5 fibroblasts, whereas the MIF inhibitor MIF098 or the CD74-blocking antibody Milatuzumab blunted this pro-migratory effect (Supplementary Figure S9H and I). By contrast, when MRC-5 cells were pre-differentiated with TGF-β1 into myofibroblast-like cells, activated B cells no longer enhanced migration (Supplementary Figure S9I), supporting a model in which B cell–derived MIF promotes the repositioning of GC-sensitive fibroblasts but is ineffective once fibroblasts acquire a TGF-β1–driven, apoptosis-resistant myofibroblast-like state. These orthogonal data support a model in which B cell–derived MIF can promote repositioning of GC-sensitive fibroblasts within OP niches, aligning with the observed spatial proximity of IGHD + B cells and fib_myo around fibroblastic plugs.

### A pathological fibroblast-epithelial niche drives irreversible remodelling in IPF

**Supplementary Figure S10:**
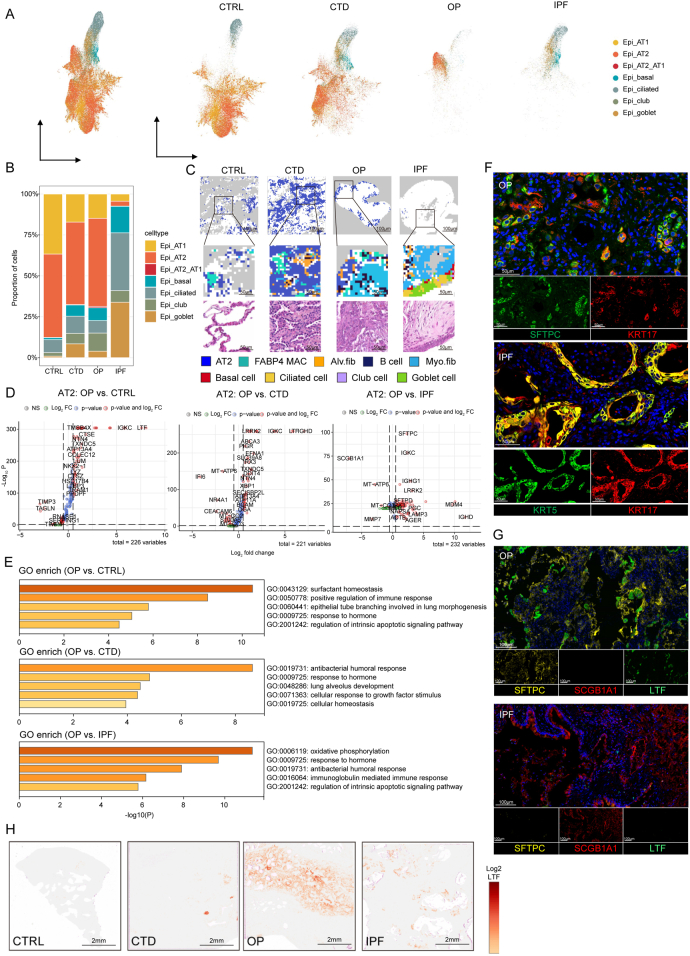
Distinct transcriptomic signature of AT2 may delineate the pathogenic mechanisms underlying organising pneumonia (OP). A) Uniform manifold approximation and projection (UMAP) plots of seven types of epithelia in the lung tissues of controls (CTRL) and patients with connective tissue disease-associated interstitial lung disease (CTD-ILD), organising pneumonia (OP), and idiopathic pulmonary fibrosis (IPF). B) Fractions of epithelial subtypes in spatial transcriptomic (ST) profiling from the CTRL, CTD-ILD, OP, and IPF groups. C) Spatial topography of the AT2 spots containing key cellular constituents in the alveolar niche. Scale bars, 100 μm and 50 μm (magnified image). D) Differential expression analysis comparing OP with CTD-ILD, IPF, and CTRL high-definition (HD) ST data per area to identify the significant differentially expressed genes (DEGs). The representative genes are labelled. Dashed lines: BH-adjusted *P* adj = 0.05 and log 2 FC = 0.25. E) Accessible gene ontology terms for AT2 from the upregulated DEGs from the comparisons of OP with CTRL, CTD-ILD, and IPF (BH-adjusted q < 0.05). F) Immunofluorescence (IF) shows that OP alveolar epithelium (SFTPC+, green) expresses KRT17 (red), whereas IPF bronchial basal cell (KRT5+, green) expresses KRT17 (red); Scale bar, 50 μm. G) IF shows that OP alveolar epithelium (SFTPC+, yellow) expresses LTF (green), whereas IPF bronchial epithelium (SCGB1A1+, red) lacks LTF; Scale bar, 100 μm. H) Spatial expression of LTF in the four groups; Scale bar, 2 mm.

Crucially, OP Fib_alv spots maintained physical segregation from fib_myo clusters, indicating compartmentalised repair microenvironments. Spatiotemporal analysis of epithelial-cell states revealed distinct alveolar maintenance mechanisms in OP. UMAP clustering identified preserved AT2 cell-spots populations in OP lesions, in contrast to predominant bronchiolisation signatures of IPF (Supplementary Figure S10A–B). Although AT2 cell-spots were also preserved in CTD-ILD, bronchial cell populations were apparent (Supplementary Figure S10B). Spatial mapping revealed OP-specific AT2 cell niches proximal to Fib_alv and FABP4+ alveolar macrophage-spots, whereas AT2 cell-spots were scarce in IPF (Supplementary Figure S10C). OP AT2 cell-spots exhibited infection-responsive plasticity, marked by LTF upregulation, activation of responses to hormone pathways, surfactant homoeostasis, and negative regulation of the intrinsic apoptotic pathway (Supplementary Figure S10D and E). In OP, KRT17 was predominantly upregulated in SFTPC + AT2 cells, whereas in IPF it was chiefly expressed in KRT5+ basal cells, consistent with KRT17's role in stress-induced epithelial remodelling, proliferation. These characteristics were validated by spatial transcriptomics and IF, suggesting that infection may be a critical trigger in OP pathogenesis (Supplementary Figure S10F–H).

### A macrophage-dominated niche characterises CTD-ILD

**Supplementary Figure S11:**
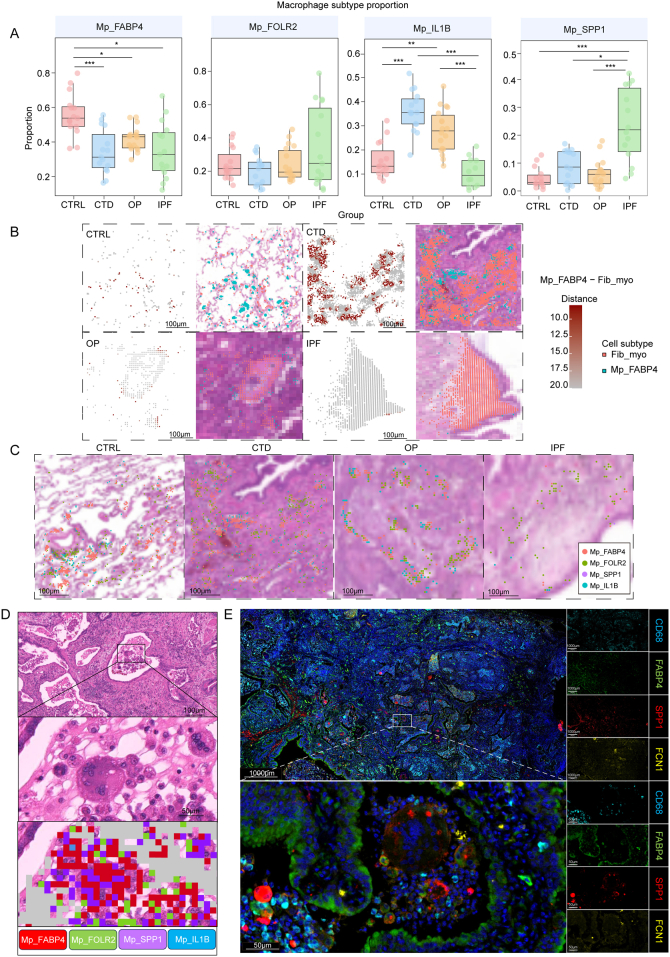
Macrophages are hallmark pathological cells in connective tissue disease-associated interstitial lung disease (CTD-ILD). A) Fractions of macrophage subtypes in spatial transcriptomic (ST) profiling from CTRL, CTD-ILD, OP, and IPF groups. Group comparisons were performed using pairwise Wilcoxon rank-sum tests with Benjamini-Hochberg adjustment; median and interquartile range (IQR; [Q3−Q1]); ∗*P* < 0.05; ∗∗*P* < 0.01; ∗∗∗*P* < 0.001; n = 5 (CTRL, CTD-ILD, IPF groups); n = 6 (OP group). B) Distance from mp_FABP4 to Fib_myo in the four groups. Scale bar, 100 μm. C) Spatial distribution of four macrophage subtypes in the four groups. Scale bar, 100 μm. D) ST reveals the cellular composition of multinuclear giant cells in the CTD-ILD group. Scale bars, 100 μm and 50 μm (magnified image). E) Multinucleated giant cells are SPP1, FABP4 positive. Scale bars, 1000 μm and 50 μm (magnified image).

scRNA-seq analysis revealed that macrophages constituted the majority of cells in CTD-ILD (Fig. 2A), with FABP4+ MAC and IL1B + MAC being the dominant subtype (Fig. 2B). Further analysis of the spatial composition of macrophage-spots revealed that FABP4+ MAC dominated in CTRL and OP, CTD-ILD exhibited the highest proportion of IL1B + MAC-spots, while SPP1+MAC spots increased in IPF (Supplementary Figure S11A). Augmentation in the number of macrophages, along with the compression of the alveolar space in CTD-ILD, positioned macrophages in closer proximity to the myofibroblasts adjacent to the alveolar layer (Supplementary Figure S11B). Moreover, spatial transcriptomics revealed distinct spatial distribution patterns of macrophage phenotypes in CTD-ILD, OP, and IPF compared with the controls (Supplementary Figure S11C). Multinucleated giant cells, formed by macrophage fusion, were observed (Supplementary Figure S11D). These cells were exceptionally large, with diameters ranging from approximately 50 to 250 μm. Spatial transcriptomic analysis and IF indicated that multinucleated giant cell-spots were primarily composed of FABP4+ and SPP1+ MAC-spots (Supplementary Figure S11E), highlighting the fusion characteristics of macrophages in chronic fibrotic progression.

## Discussion

This spatial transcriptomic study provides a systematic comparison of fibroblast heterogeneity among OP, CTD-ILD, and IPF, supporting a model where clinical trajectories are dictated by the local cellular niche rather than fibroblast–intrinsic properties alone. In reversible OP, an ‘alveolar-protective’ niche—co-inhabited by IGHD + B cells and plastic AT2 cells—programs myofibroblasts toward a glucocorticoid-sensitive, apoptosis-primed state with restricted COL1A2 expression, thereby enabling alveolar restoration. Conversely, in progressive IPF, the loss of this regenerative context and its replacement by a ‘bronchiolised’ epithelial niche creates a pro-fibrotic sanctuary that locks fibroblasts into an apoptosis-resistant, persistently activated state, driving irreversible scarring. CTD-ILD occupies a macrophage-rich and immune-skewed domain, maintaining fibroblasts in an oxidative, pro-inflammatory phenotype. Complementing these spatial findings, multiplex immunofluorescence delineates immune–epithelial–stromal niche features across conditions and stages—early repair in OP, recurrent/exacerbating and late fibrotic remodelling in IPF, and chronic inflammation with episodic flares in CTD-ILD. These findings extend prior single-cell studies by resolving fibroblast–microenvironment relationships and align with the biological sequence of injury, organisation, and repair emphasised by ATS/ERS statements,^2^^,^^13^^,^^14^^,^^20^^,^^30^^,^^31^ suggesting that these conditions represent a divergence in spatial programming: successful resolution in OP, stromal autonomy in IPF, and sustained immune-mediated injury in CTD-ILD.

Building on this framework, our data reveal that spatial niches dictate disease-specific pathologies. In OP, fibroblasts co-localise with B-cells and LTF^+^ AT2 cells, sustaining alveolar integrity via antimicrobial pathways, consistent with infection-driven pathogenesis,^32^ unlike IPF which drives bronchiolisation. Phenotypically, OP fib_myo form GC-sensitive niches with B-cells via MIF-CD74 signalling, exhibiting dual apoptotic competence via mitochondrial and death–receptor pathways. GC therapy amplifies this state by upregulating stress and resolution genes, culminating in caspase activation. Conversely, IPF fib_myo localise to bronchiolised regions within COL1A2^+^ fibrotic niches resistant to GC-induced apoptosis. Although OP exhibits a marked increase in the number of CTHRC1^+^ myofibroblasts, the per-cell expression intensity decreases, distinguishing it from IPF where the elevation is driven by higher detection without increased intensity; this “more-but-weaker” profile highlights a key feature of fibroblasts in reversible fibrosis.^22^^,^^33^^,^^34^^,^^35^ The abundance of GC-sensitive fibroblasts in OP and their proximity to reparative niches suggest a preserved apoptosis-driven resolution capacity.

**Supplementary Figure S12:**
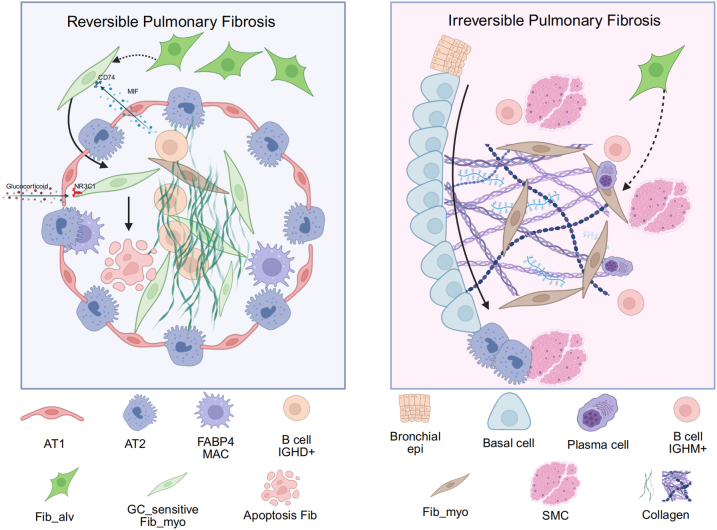
Cellular landscapes in reversible versus irreversible pulmonary fibrosis. Using High-Definition Visium, we performed a comparative analysis of reversible versus irreversible pulmonary fibrosis. In reversible fibrosis, glucocorticoid-responsive, apoptosis-prone fibroblasts—together with IgD + B cells—maintained a dynamic equilibrium around largely preserved alveoli with limited collagen deposition, thereby enabling tissue repair. *In vitro* experiments confirmed that IgD + B cells promote the migration of susceptible MRC-5 fibroblasts via the MIF–CD74 axis. By contrast, irreversible fibrosis was characterised by myofibroblasts that were poorly responsive to glucocorticoids and displayed anti-apoptotic features. Excessive accumulation of fibroblasts and collagen led to tissue stiffening, accompanied by disorganised bronchial epithelium, ultimately indicating irreversible architectural damage.

Our spatial data place IgD + B cells within alveolar niches where they co-localise with GC-responsive, apoptosis-primed myofibroblasts. Mechanistically, membrane IgD denotes an antigen-experienced, hyper-responsive B-cell state whose hinge region tunes BCR signalling thresholds, enabling rapid responses to persistent antigens.^36^ IgD engagement can amplify pro-inflammatory output from human mononuclear cells and IgD Fc receptor signalling can directly activate stromal cells, supporting a B-cell-to-mesenchyme axis.^37^^,^^38^^,^^39^ These features fit a model in which IgD + B cells act as sentinel amplifiers that transiently escalate danger signals within preserved alveoli, helping organise a reparative, steroid-responsive niche. Consistent with this model, our *in vitro* perturbations validate that B cell–derived MIF promotes migration of GC-sensitive fibroblasts via CD74, matching the spatial co-localisation seen in early OP.^40^^,^^41^^,^^42^ Unlike perivascular/bronchiolised B-cell topologies in IPF, IgD + B cells in OP are embedded in alveolar compartments with preserved basement membrane, a spatial context favouring reversibility. Within an epithelial context marked by LTF + AT2 cells and intact surfactant programs, this loop could recruit myofibroblasts into alveoli yet maintain a GC-sensitive, apoptosis-competent state (elevated NR3C1, DDIT4, BCL2L11; coordinated caspase activation) in OP versus IPF. B cell–derived MIF drives CD74-mediated migration in GC-responsive fibroblasts within early OP, but this effect is blunted by TGF-β in late, fibrotic niches. This framework enriches the understanding of B-cell multi-faceted roles across ILD progression.^9^^,^^10^^,^^12^^,^^16^^,^^17^^,^^18^^,^^19^ In CTD-ILD, multiplex IF (CD68, FCN1, FABP4, SPP1) delineates multinucleated macrophage composition within macrophage-dominated niches, reflecting chronic inflammation with recurrent acute exacerbations. This late pro-fibrotic macrophage phenotype aligns with the immune-skewed, oxidative fibroblast state we observe, and may help explain clinical trajectories in CTD-ILD (Supplementary Figure S12).

Three limitations warrant consideration. First, the cross-sectional design limits the resolution of temporal dynamics. While our staging is grounded in established criteria, future longitudinal spatial profiling is needed to confirm the proposed trajectories. Second, HD Visium may underestimate senescent populations in low-cellularity fibrotic regions. Third, we deconvolved OP spatial data using a non-OP scRNA-seq reference, reflecting ethical and practical constraints. OP is diagnosed post hoc, glucocorticoids act rapidly, and fresh biopsies for atlas building are unjustified. Lacking public OP scRNA-seq, we leveraged conserved lung lineages and OP-like features in CTD-ILD and IPF. Validation covered RCTD confidence, marker concordance, replicate reproducibility, IF, and functional signatures.

In conclusion, our comparison of OP, CTD-ILD, and IPF defines fibrotic lung disease as a disorder of “cellular niche dysregulation.” Currently, no pharmacotherapy effectively reverses the apoptosis-resistant phenotype of IPF fibroblasts to restore the GC-sensitive state of OP. Our data suggest that while B-cell accumulation in OP facilitates adaptive repair, the dysfunctional niche in IPF locks fibroblasts into irreversible senescence. Future strategies must move beyond broad immunosuppression toward ‘senolytic’ or ‘rejuvenating’ therapies capable of reprogramming the epigenetic landscape to restore GC sensitivity. Indeed, early clinical pilots of senolytics have shown promise in alleviating physical dysfunction in patients with IPF, validating the potential of targeting senescence pathways.^43^^,^^44^^,^^45^^,^^46^ While challenging, targeting these niche-driven senescence pathways offers a rational, albeit difficult, path toward overcoming the barrier to resolution in progressive fibrosis.

## Contributors

LX, HC, JC, and WC conceived the study. LS, YY, YF, YL and QL contributed to conceptualisation, study design, methodology, data collection, validation, and formal analysis. MZ, CY, JC and WC provided the lung tissue samples used in this study. LS, YF, RS and WC contributed to formal analysis and methodology. YY, YL, DS and QL contributed to formal analysis, visualisation, and manuscript writing. HC and LX contributed to investigation, resources, data curation, supervision, project administration, and funding acquisition. All authors have read and approved the final version of the manuscript prior to submission.

## Data sharing statement

Data availability: Source data are provided in this study. The accession numbers for the raw HD-visium reported in this paper have been deposited in the Genome Sequence Archive for Humans (https://ngdc.cncb.ac.cn/gsa-human/) under the accession number HRA011271. Huaiyong Chen and Lixin Xie had full access to all the raw data generated in this study. They independently verified the data against laboratory records and analysis outputs to confirm accuracy and completeness. All authors have read and approved the final version of the manuscript prior to submission.

Code availability: The software codes are publicly available at the following links: https://github.com/songlicheng666/Microenvironmental-Niches-in-Reversible-versus-Progressive-Lung-Fibrosis; https://github.com/yifanfu01/stGrads.

## Declaration of interests

The authors have declared that no competing interest exists.

## Figures and Tables

**Original Western blot images:**
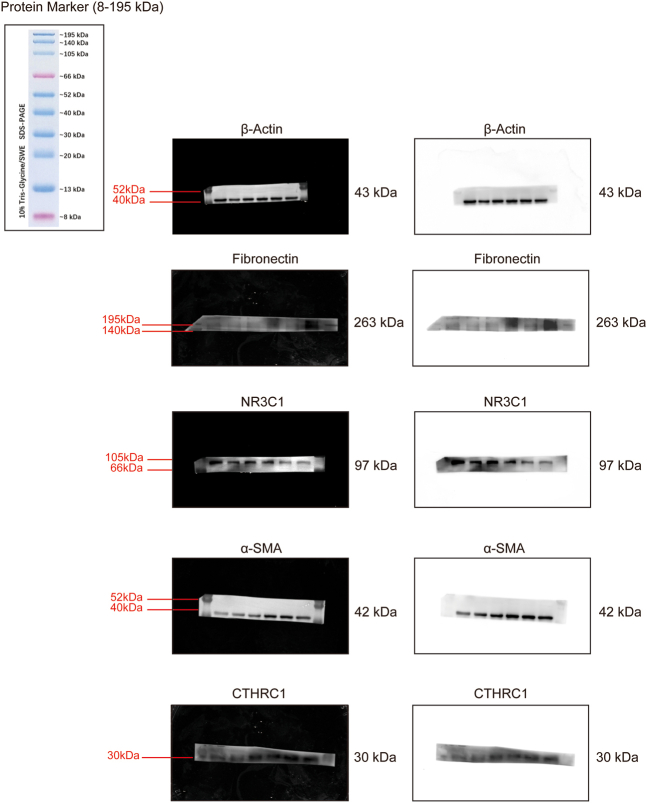


## Appendix B Supplementary data

Supplementary Table S1 Sample and demographic information. Abbreviations: ASS-ILD = antisynthetase syndrome–associated interstitial lung disease; CTD-ILD = connective tissue disease–associated interstitial lung disease; CTRL = control; HD Visium = high-definition Visium spatial transcriptomics; IF = immunofluorescence; IHC = immunohistochemistry; IPF = idiopathic pulmonary fibrosis; OP = organising pneumonia; RA-ILD = rheumatoid arthritis–associated interstitial lung disease; scRNA-seq = single-cell RNA sequencing; SLE-ILD = systemic lupus erythematosus–associated interstitial lung disease; SSc-ILD = systemic sclerosis–associated interstitial lung disease.

Supplementary Table S2 Comparison of Clinical and Pathological Features Between Surgically Resected and Biopsy-Confirmed Steroid-Responsive OP Cohorts. Data are presented as n (%), mean and standard deviation (SD), or median and interquartile range (IQR; [Q3−Q1]). P-values were calculated using the Mann–Whitney U test for continuous variables (Age) and Fisher's exact test for categorical variables (Sex, Clinical Diagnosis, Sample Site).

Supplementary Table S3 Master list of cell lines, antibodies, and key reagents. Abbreviations: ACTA2 = alpha-smooth muscle actin (α-SMA); CD19 = cluster of differentiation 19; CD68 = cluster of differentiation 68; CD74 = cluster of differentiation 74; CTHRC1 = collagen triple helix repeat containing 1; FCN1 = ficolin 1; FABP4 = fatty acid binding protein 4; IF = immunofluorescence; LTF = lactotransferrin; MAC = macrophage; MIF = macrophage migration inhibitory factor; MRC-5 = human fetal lung fibroblast cell line; NR3C1 = nuclear receptor subfamily 3 group C member 1 (glucocorticoid receptor); PARP1 = Poly(ADP-ribose) polymerase 1; PBMCs = peripheral blood mononuclear cells; SFTPC = surfactant protein C; SCGB1A1 (CC10/CC16) = secretoglobin family 1A member 1 (club cell secretory protein); SPP1 = secreted phosphoprotein 1 (osteopontin, OPN); TGFB1 = transforming growth factor beta 1 (TGF-β1); WB = western blot.
