# Supplementary material for: Microenvironmental niches dictate divergent fibroblast fates in reversible versus progressive lung fibrosis
Source: eBioMedicine. 2026 Jan 30;124:106142. doi: 10.1016/j.ebiom.2026.106142 (PMC12878701; doi:10.1016/j.ebiom.2026.106142)

MRC-5 cells STR report

Method: An appropriate amount of "MRC-5" cells (Cell number PC-H2025091911,1×10^6) were used TIANamp Genomic DNA Kit to extract DNA, 20 STR loci and gender identification loci were amplified by Microreader™21 ID System, PCR product detection was performed by GenReader 7010 genetic analyzer, detection results were analyzed by GeneMapper Software6 (Applied Biosystems), and compared with Cellosaurus databases.

Experimental result:

1. The results of negative and positive control were correct.
2. The genotyping results of STR locus of "MRC-5" cell line is shown in the following table.

Conclusion:

1. The genomic DNA of "MRC-5" cell line is clear and the result of genotyping is good.
2. The results of STR typing showed that no cross contamination of human cell was found in the cell line of "MRC-5" cell line.
3. The DNA typing of the cell line was 100.00% matched with the cell type in the cell bank, and the cell line name was MRC-5.

Appendix I: The genotyping results of STR locus of "MRC-5" cell line.

STR Loci	Sample: PC-H2025091911	Database：MRC-5
Amelogenin	X, Y	X,Y
CSF1PO	11,12	11,12
D2S1338	20	20
D3S1358	15,17	15,17
D5S818	11,12	11,12
D7S820	10,11	10,11
D8S1179	13	13
D13S317	11,14	11,14
D16S539	9,11	9,11
D18S51	15,21	15,21
D19S433	14,15	14,15
D21S11	31.2	31.2
FGA	21,23	21,23
PentaD	12	12
PentaE	12,16	12,16
TH01	8	8
TPOX	8	8
vWA	15	15
D6S1043	11,19	11,19
D12S391	20,22	20,22
D2S441	10,11	
The Cellosaurus database has a matching rate of 100.00%, The number of matched bits is 19 (https://www.cellosaurus.org/index.html)		

Note:

1. According to the cell STR identification standard established by the International Cell Line Authentication Committee (ICLAC), when the matching degree of cell lines is ≥80%, they are considered to be correlated, that is, derived from common ancestral cells; The matching degree is between 55% and 80%, and the correlation needs to be further verified. Less than 55% indicates no correlation between the two.
2. The effective peak of the map was the real PCR band; Small peaks and nonspecific bands were ignored in the calculation.
3. The default Cellosaurus STR data comparing the results, the data sources include ATCC, DSMZ, JCRB cell library and documents and records, such as https://www.cellosaurus.org/index.html database entry.

Appendix II: The genotyping results of STR locus of "MRC-5" (Cell number PC-H2025091911) cell line.

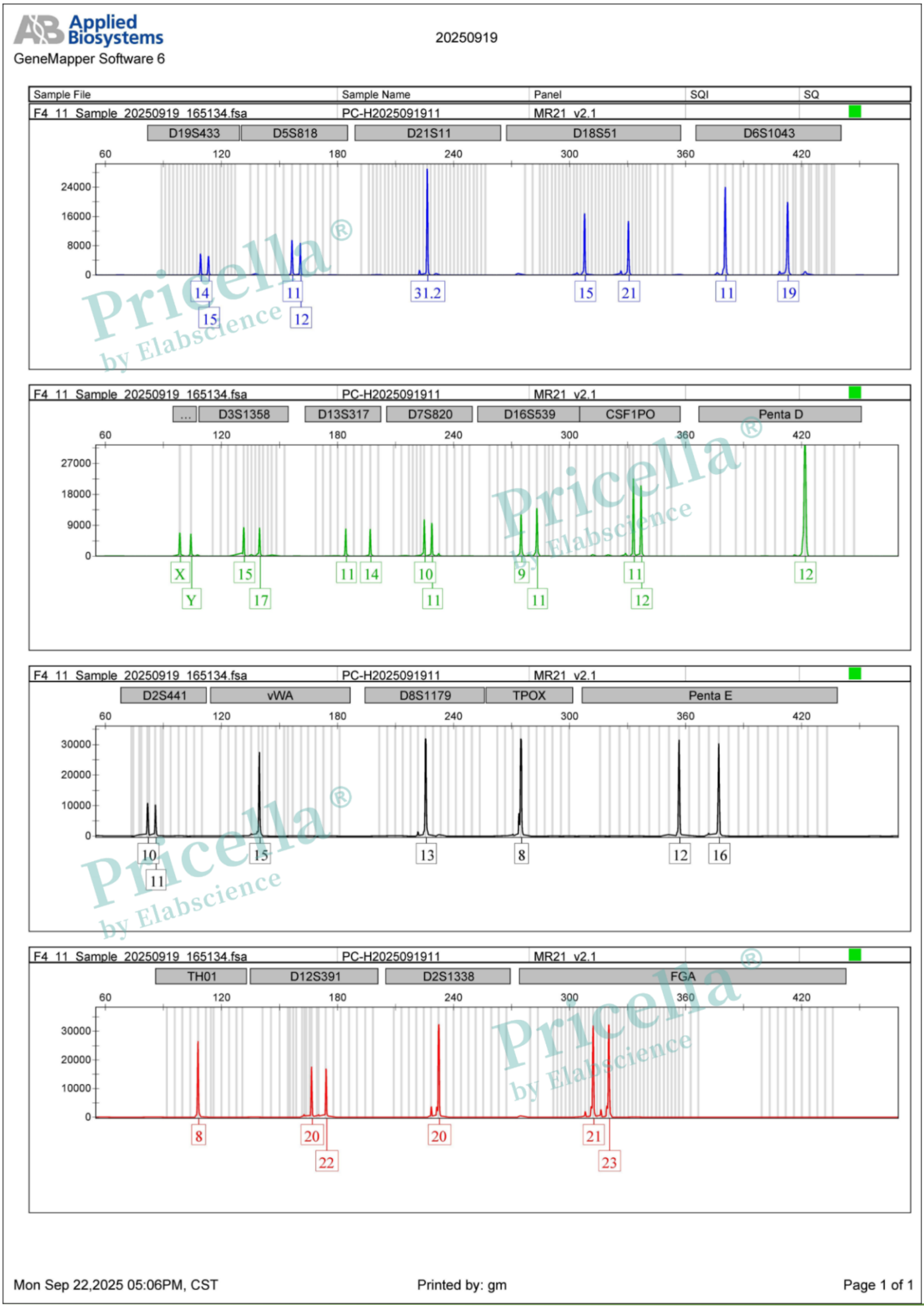

Supplement: Supplementary Material 1 [file mmc4.pdf]
