# Supplementary material for: Microenvironmental niches dictate divergent fibroblast fates in reversible versus progressive lung fibrosis
Source: eBioMedicine. 2026 Jan 30;124:106142. doi: 10.1016/j.ebiom.2026.106142 (PMC12878701; doi:10.1016/j.ebiom.2026.106142)
Supplement: Supplementary Material [file mmc5.docx]

**Supplementary Methods**

**Sample Preparation of Human Lung Tissues**

CTD-ILD and IPF lung tissues were obtained from patients who underwent lung transplantation. Patients with CTD were selected based on a diagnosis of CTD-ILD and had undergone lung transplantation due to bilateral pulmonary fibrosis. OP lung tissues were obtained from surgical lobectomies. Prior to surgery, all patients with OP exhibited unexplained pulmonary nodules or lung consolidation and were subsequently diagnosed with OP through pathological examination post-surgery. During the follow-up period, which included tracing and aetiological analysis, we determined that prolonged infection might have been a significant contributing factor to the development of OP. Healthy lung tissue was obtained from deceased donors with no known lung diseases. All human control, CTD-ILD, and IPF lung tissues were collected by resection from the peripheral lung. Detailed clinical information for all patients is provided in Supplementary Table S1.

Building on the above tissue sources, we conducted a retrospective cohort study to assess whether imaging–pathology features observed in surgically resected OP are generalisable to typical steroid-responsive OP encountered in routine care. The surgical cohort included 10 patients who underwent lung resection for OP lesions, and the comparator cohort comprised 10 patients with percutaneous biopsy–confirmed OP who subsequently received systemic glucocorticoids with documented clinical or radiological improvement. To contextualise disease specificity beyond OP, we assembled a small idiopathic pulmonary fibrosis reference set of five cases as a negative control. All analyses were approved by the institutional review board and followed a pre-specified analytic plan emphasizing blinded assessments, reproducibility metrics, and sensitivity analyses.

**Imaging Assessment of Surgically Resected OP and Percutaneous Biopsy–Confirmed OP**

Thoracic CT scans were independently reviewed by two fellowship-trained thoracic radiologists who were blinded to cohort assignment and clinical outcomes. At the lesion level, readers scored pre-defined, reproducible features on a four-point 0–3 scale denoting absent, mild, moderate, or marked expression. The features included the proportion of ground-glass components, margin sharpness, presence of air bronchograms, the proportion of consolidation, involvement across fissures or lobar boundaries, and signs consistent with bronchiolization. To capture a consolidated radiological signature of organizing pneumonia, we derived a composite OP Imaging Score that increased with ground-glass and air bronchogram prominence and decreased with consolidation using equal weights. Disagreements after independent review were resolved in a consensus session, and inter-reader agreement was quantified using weighted kappa with a priori acceptability set at 0.60 or higher.

**Histopathology Annotations**

For standardized spatial annotation and downstream visualization, H&E-stained sections were manually labelled by experienced thoracic pathologists to identify typical regions representing distinct lung pathologic contexts: ‘blood vessel’ ($), ‘large airway’ (Ω), ‘organizing pneumonia (OP)’ (&), ‘fibroblastic foci of UIP’ (▲), ‘diffuse lung injury’ (%) and ‘within normal limits (alveolar)’ (@). ‘Diffuse lung injury’ referred to areas characterised by conspicuous inflammatory cell aggregates, thickened and fibrotic septa, intraalveolar oedema/haemorrhage, and alveolar collapse or hyperinflation. ‘Fibroblastic foci’ annotations were reserved for UIP-type lesions and were based on microscopic criteria including epithelial hyperplasia/bronchiolization, subepithelial spindle-cell tufts with increased collagenous matrix, and nuclear crowding/shape consistent with UIP foci. These annotation classes were used solely to localize representative regions for cross-disease comparison and do not reclassify OP cases as UIP. Subsequently, sections from 5 CTD-ILD, 5 IPF, 6 OP, and 5 control individuals containing these representative regions were processed in the HD Visium workflow.

**Histopathology Assessment of Surgically Resected OP and Percutaneous Biopsy–confirmed OP**

Haematoxylin and eosin–stained slides were analysed using ImageJ (version 1.53t; National Institutes of Health, Bethesda, MD, USA) with the Segmentation plugin (Trainable Weka Segmentation). For each case, three histopathological regions of interest were pre-defined: (i) fibroblastic plugs, (ii) AT2-retention, and (iii) inflammation. Region classifiers were trained on representative annotations using multi-feature inputs, including gaussian blur, sobel filter, hessian, difference of gaussians, and membrane projections. After supervised learning, the trained model was applied to each H&E slide to compute the percentage area occupied by each region (fibroblastic plugs, AT2-retention, inflammation). Independent of the automated readout, two pulmonary pathologists, blinded to imaging, cohort, and outcomes, performed review and correction of the region maps; corrected percentages were then recorded as the final scores for each region. Inter-reader reproducibility was summarized with weighted kappa using a prespecified adequacy threshold (κ ≥ 0.60). Primary between-cohort analyses were designed to test similarity of the OP pathologic pattern between surgically resected and biopsy-confirmed OP at the pattern level based on these three region scores, rather than to establish temporal staging differences.

**OP Staging Criteria and Blinded Pathology Review**

We implemented an H&E- and IF-based, ROI-level staging system **which reflects** the biological sequence from inflammation-dominant to fibroplasia and resolution.

Early inflammatory stage: alveolar spaces show dense infiltration by inflammatory cells (lymphocytes, macrophages, and neutrophils) with increased nuclear density and amorphous/proteinaceous exudates; organized spindle-shaped fibroblastic proliferations and collagen deposition are absent.

Middle fibro-inflammatory stage: fibroblasts/myofibroblasts expand and form fibroblastic buds that partially envelop resident inflammatory cells, consistent with immature granulation tissue; inflammatory cells are still present but increasingly embedded within fibroblastic matrices.

Late resolution/fibrotic stage: fibroblasts undergo contraction/senescence with accompanying collagen deposition; immune cells within fibroblastic plugs largely disappear; alveolar integrity is restored with re-opened airspaces and continuous epithelial lining. Apart from resident alveolar macrophages, intra-alveolar lymphocytes/neutrophils are minimal or absent.

Two pulmonary pathologists independently staged all ROIs based on the above criteria, blinded to group labels and molecular data, and reached complete concordance in their assignments. We restricted inferential analyses to ROIs from ST data with concordant expert labels and performed all statistics at the donor level (ROI→donor aggregation) to avoid pseudo-replication.

**Single-cell RNA Sequencing**

For control, CTD-ILD, and IPF lung samples for single-cell RNA sequencing, fresh samples were washed with PBS and minced into small pieces. These tissues were digested with collagenase IV (1 mg/ml; Sigma, cat. no. C1889), Dispase II (1 mg/ml; Roche, cat. no. SCM133), and DNase I (0.1 mg/ml; Sigma, cat. no. DN25) with continuous stirring at 37 °C for 1 h. Single-cell suspensions were filtered through a 100 μm strainer. All single-cell suspensions were washed twice with PBS before further experimentation. All cells were collected and libraries were constructed according to 10x Genomics single cell sequencing kit following the recommended protocol, and sequenced on the Illumina platform. Detailed clinical information for all patients is presented in Supplementary Table S1.

**ScRNA-seq Data Processing and Clustering**

Raw gene expression matrices were merged and processed by Seurat (1). To exclude doublets, we first filtered out cells of low quality based on three metrics: (1) cells with fewer than 200 genes or >6000 genes. (2) cells with fewer than 500 or >30000 unique molecular identifiers (UMIs). (3) Cells with mitochondrial gene percentages exceeding 20%. Then, “DoubletFinder” package was utilized to estimate potential doublets in each sample and potential doublets were removed (2). The integration of different sample batches was performed using the harmony method (3). Scaled data after normalization were used for principal component analysis (PCA) using 2000 highly variable genes. The first 15 principal components were utilized for UMAP embedding, with the first 2 principal components serving as UMAP initialization. Differentially expressed genes were identified using the “FindAllMarkers” to functionally characterise the clusters. For trajectory analysis, the “Slingshot” R package (4) was employed to perform pseudotime ordering and infer the state evolution of myofibroblasts, lipofibroblasts and alveolar fibroblasts. Gene Ontology (GO) enrichment analysis was performed using Metascape (version 3.5, https://metascape.org/), with biological processes considered significant at p < 0.01. GO terms were visualised and scored using the Seurat package.

**Trajectory Analysis Predicts Differentiation of Alveolar Fibroblast, Lipofibroblast and Myofibroblast**

To evaluate potential lineage relationships within alveolar fibroblast, lipofibroblast and myofibroblast, pseudotime analysis was performed using Slingshot (4-6). First, specific cell types (Fib_alv, Fib_myo, and Fib_lip) were selected for analysis, and data preprocessing was performed using the Seurat package, including PCA and UMAP dimensionality reduction, KNN graph construction, and clustering analysis. Trajectory inference was performed on the UMAP results using the Slingshot package, with Fib_alv defined as the starting point. Pseudotime trajectories were visualised, and dynamic changes in gene expression along the trajectory were analysed using the TradeSeq package. During trajectory inference, highly variable genes (top 2000) were selected, and models were fitted (fitGAM) using pseudotime and cell weights. Subsequently, genes associated with the trajectory were identified through association testing (associationTest). The pseudotime trajectory was divided into multiple intervals, and heatmaps were used to visualise gene expression patterns across different pseudotime intervals. Additionally, several genes of interest (e.g. CTHRC1, COL1A2, and PDGFRA) were selected, and heatmaps annotated with these genes were generated using the ComplexHeatmap package to further explore their dynamic changes along pseudotime.

**Cell-cell Communication Analysis**

CellChat was employed to deduce and scrutinise intercellular communication networks based on its ligand-receptor interaction databases (http://www.cellchat.org/)(7).Interactions were discerned and assessed based on the expression levels of ligands and receptors differentially overexpressed in each cell group (*p* < 0.05). To assess the differential interaction strength between groups, intercellular communication networks were inferred and merged using the "mergeCellChat" function. The interaction strength was quantified using the "computeCommunProb" function. A heatmap was generated using "netVisual_heatmap" to visualise changes in intercellular communication, with group comparisons specified via the "comparison" parameter. The relative interaction strength was represented by a gradient colour scale. Comparisons of communication probabilities in ligand-receptor pairs regulated by specific cell populations were made using the "netVisual_bubble" function, configured with the compare parameter.

**ST Using the Visium HD Spatial Gene Expression Platform**

According to the Visium HD FFPE Tissue Preparation Handbook (CG000684, 10x Genomics), formalin-fixed and paraffin-embedded (FFPE) slides, and RNA quality assessment of FFPE tissue blocks was performed by calculating the percentage of total RNA fragments >200 nucleotides (DV200) of RNA extracted from tissue sections. For high RNA quality, DV200 > 30% was required. Next, FFPE tissue sections were placed on blank slides for deparaffinization, HE staining, and imaging. Based on the Visium HD Spatial Gene Expression Reagent Kits User Guide (CG000685), probe hybridization, probe ligation, Visium HD slide preparation, probe release, extension and probe-based library constructions were carried out. The libraries were sequenced on an Illumina NovaSeq X Plus-25B with paired-end reads. The tissue slide within the Visium HD tissue cassette was then prepared for loading onto the CytAssist instrument. It underwent human probe hybridization (10x Genomics, 1000466) and a posthybridization wash to remove unbound probes, followed by probe ligation and subsequent wash. The Visium HD slide containing the probe capture areas was prepared. Both the Visium HD slide and tissue slide were loaded onto the CytAssist instrument to enable probe release and capture. The probe-based library was constructed using Dual Index Plate TS Set A (10x Genomics, 1000251) with a sample index PCR cycle number of 16, as determined by the Cq value from qPCR (Applied Biosystems QuantStudio 5). A final cleanup step with SPRIselect reagent was followed by storage at −20 °C until sequencing. Library quality control was performed on an Agilent TapeStation 4200. Next-generation sequencing was carried out on Illumina’s NovaSeq X platform using a 10 billion read, 300 cycle flow cells. Minimum sequencing recommendations were met using the product of tissue coverage percentage estimations and a constant maximum read figure of 275 million read pairs. Optimal cluster density was achieved with a lane loading concentration of 235 pM. All steps were performed according to the instructions found in 10x Genomics’ Demonstrated Protocol CG000685. A high-resolution image was aligned using the Visium HD Manual Alignment tool on Loupe Browser (v8.0.0; 10x Genomics). Visium HD sequence data, high-resolution images, and alignment files were processed using the count function of Spaceranger (3.0.0; 10x Genomics) with default parameters. The reads were mapped against the GRCh38-2020-A reference. Loupe Browser (v8.0.0) was used to visualise gene expression over H&E images for the Visium HD data.

**Selection of ROI Regions**

In the context of HD Visium spatial transcriptomics, each dataset generated approximately 600,000 spots. Considering the unique sac-like structure of alveoli, which contains a large number of cavities, and the presence of fibrotic regions that lead to reduced gene counts in certain spots, these factors may adversely affect the overall analysis. To address this, we conducted characteristic selection of spots for each sequencing sample with the assistance of pathology experts. Specifically, for the control group, ROI regions were selected to include at least 80% of alveolar structures, as well as the bronchioles and surrounding vascular structures. In the CTD-ILD group, ROIs were chosen to encompass areas of diffuse lung injury (including inflammatory cell infiltration and alveolar oedema) and regions of interstitial thickening, along with adjacent bronchial and major vascular structures. For the OP group, given the characteristic pathological features of intra-alveolar fibrotic plugs, ROIs were selected to include entire alveolar structures surrounding the lesions. In the IPF group, due to the presence of extensive fibrosis, ROIs were selected to include typical fibroblast foci and surrounding epithelial structures. Additionally, alveolar bronchiolization, a hallmark pathological feature of IPF, was included in the ROI selection. Using these criteria, 3 characteristic ROI regions were selected for each sample, resulting in a total of 63 ROIs. Each ROI was extracted as an independent Seurat object and subsequently merged for downstream analysis. ROIs were treated as technical/within-donor units for preprocessing and visualization; inferential statistics and DE testing were performed using pseudobulk or donor-level aggregation as specified below.

**Cell Type Decomposition and Visium HD Resolution, Analysis Unit, and Quality Control (QC)**

Visium HD data were analysed at 8 µm binning following 10x Genomics recommendations and the Seurat Visium HD vignette. We loaded 8 µm assays via Load10X_Spatial and restricted analyses to tissue-covered barcodes (filter.matrix=TRUE; tissue_positions). Per-sample QC required nFeature_Spatial ≥ 50, nCount_Spatial ≥ 100, and percent.mt ≤ 20% (computed via PercentageFeatureSet, pattern “^MT-”). Each sample was normalised independently with SCTransform, and integrated using SCT anchors (SelectIntegrationFeatures=500; PrepSCTIntegration; FindIntegrationAnchors; IntegrateData). Clustering and spatial visualization were performed on 8 µm bins (Spatial.008um) using standard Seurat workflows; tissue domains were optionally identified with BANKSY. We explicitly note that we did not perform image-based cell polygon segmentation; all “bin-level” references denote near-bin-scale bins at 8 µm resolution. Spatial boundaries are thus defined by bin centroids and tissue masks from scalefactors_json.json/tissue_positions, rather than by per-cell polygons.

**RCTD Deconvolution and Integration Quality Assessment**

To obtain the distribution of cell groups in the spatial region, we used the integrated scRNA-seq dataset as reference to perform cell type decomposition in histological structures of the ST slide using the RCTD method (8). We built the scRNA-seq reference with 35 cell subtypes, replaced special characters in labels, and constructed the Reference (FlexRef, CTRef, nUMI) object using spacexr. Visium HD 8 µm-binned data were loaded from Cell Ranger outputs, and bin centroids were used to create a SpatialRNA object. Before deconvolution, we performed quality control by filtering out low-quality bins using thresholds of counts < 10, UMI < 10, and UMI_min_sigma = 10, keeping other RCTD parameters at their default settings. We ran RCTD in doublet_mode = "doublet" with default parameters on up to 12 cores. For each bin we recorded first_type and second_type as the top two reference labels, the singlet_score as the posterior weight on the top label, the min_score, and the convergence flags. Integration quality was summarized by assignment confidence using the singlet_score distribution and the margin between first_type and second_type. Spatial consistency was assessed by agreement between unsupervised spatial clusters and RCTD labels using a row-normalised confusion matrix, reporting per-cluster top-1 purity and global adjusted or normalised mutual information. Only spots with successful convergence were included. Orthogonal validation included expected spatial patterns of canonical markers, immunofluorescence and immunohistochemistry for selected lineages, and concordant spatial enrichment of GC-response and apoptosis signatures, supporting reliable deconvolution with a non-OP integrated scRNA-seq reference.

**Two-part Modelling of ST Gene Expression**

We analysed normalised bin-level expression using a two-part (hurdle) framework that separates detection from intensity among positives. For CTHRC1, we first modelled the probability of detection (expr > 0) with a logistic regression: detection ~ group (baseline = CTRL), reporting odds ratios (OR) and 95% confidence intervals (CIs). Among positive cells (expr > 0), we modelled expression intensity using log-normal via linear regression on log-transformed expression, reporting fold-change ratios and 95% CI. Coefficients were exponentiated for interpretability. We summarized overall expression per group using the product approximation E[expr] ≈ P(expr > 0) × E[expr | expr > 0], applying log-normal mean correction E[X]=exp (μ+σ^2^/2) from the fitted log-normal model. P values and confidence intervals were obtained via standard model-based inference. CTRL was used as the reference level for all contrasts. Forest plots display group-wise effect sizes on a log scale with 95% CI.

**Cell-to-cell Spatial Proximity Analysis**

Coordinate system and unit conversion. We used spot/bin centroid coordinates from HD Visium FFPE at 8 µm binning; pixel coordinates extracted via Seurat were converted to micrometres using the manufacturer’s scale (all distances reported in micrometres; where applicable legacy assays referencing spot diameter 383.63 µm were mapped accordingly).

Distance definition and computation scope. For each region of interest (ROI), we computed Euclidean centroid-to-centroid nearest-neighbour distances from source to target spot sets within the same ROI; cross-ROI distances were not considered. Source sets were high-confidence singlets (singlet_score ≥ 0.8) labelled by RCTD (doublet_mode="doublet"; default parameters), whereas target sets were defined by canonical markers and/or UCell signatures (B cells, AT2 cells, FABP4+ macrophages, capillary endothelia). A custom function calculated nearest distances between specified cell-type pairs per sample/ROI, summarizing mean, median, standard deviation (SD), and standard error of the mean (SEM), with density plots/boxplots for distance distributions.

Boundary and density controls with permutation. To mitigate edge effects, source spots closer than the analysis radius (typically 200 µm) to ROI boundaries were excluded in sensitivity analyses. To control for local density and area differences, we performed within-ROI label permutations (n=1,000) of target identities while preserving source positions, generating null nearest-neighbour distributions. We report observed-to-expected enrichment (Obs/Exp) of the median nearest-neighbour distance and permutation p-values, alongside comparisons of observed means against randomised distributions.

Statistical aggregation and inter-group comparisons. Nearest-neighbour distances were summarized at the ROI level and aggregated to donor-level medians to avoid pseudo-replication at the spot level. Inter-group analyses across CTRL, OP, IPF, and CTD-ILD used Kruskal–Wallis tests on donor-level medians with Benjamini–Hochberg correction; significant results underwent Dunn’s post hoc tests with BH adjustment to identify specific group differences. Donor-level bootstrap (1,000 replicates) provided 95% confidence intervals for median differences. Results were robust to alternative target definitions and analysis radii (25–200 µm). Visualization included boxplots for inter-group comparisons and kernel density plots for within-group distance distributions. The cell location was identified and the nearest distance was calculated by stGrad package (<https://github.com/yifanfu01/stGrads>). Briefly, the distance between one given cell type and the nearest other cell type was defined as cell-cell radial distance. The distribution of distance was exhibited by density curve. The maximum interaction distance was set as 200μm for most suitable consideration. The linear model was used in this study, the other parameters were set as default.

**Pathway Enrichment**

To avoid pseudo-replication from 8 µm bin–level measurements, differential expression (DE) and pathway enrichment were performed using a pseudobulk strategy. Briefly, for each ROI within each donor, we aggregated Visium HD raw gene counts by summing counts across all tissue-covered 8 µm bins in that ROI, yielding one pseudobulk expression profile per ROI unit. Pseudobulk count matrices were log-normalized in Seurat prior to DE testing (Wilcoxon rank-sum test). DE between groups was then computed on these pseudobulk profiles in Seurat using FindMarkers/FindAllMarkers (min.pct ≥ 0.1, logfc.threshold ≥ 0.25), with P values adjusted by the Benjamini–Hochberg method. For pathway enrichment, we used Metascape (https://metascape.org/), submitting gene symbols from each DEG list. Analyses were performed with species set to **H. sapiens** and the database restricted to GO Biological Processes, using Min Overlap = 3, P Value Cutoff = 0.01, and Min Enrichment = 1.5. Metascape performs hypergeometric testing with multiple-hypothesis correction and reports enriched terms meeting these thresholds. Enrichment results (term name, P value, enrichment score, and gene members) were exported and summarized per comparison. As complementary visualization of pathway activity, curated gene sets were scored using Seurat::AddModuleScore and visualized spatially (SpatialFeaturePlot) and across groups (VlnPlot).

**Single-cell-wise Gene Signature Scoring**

Gene sets for scoring (Apoptosis score: GOBP_APOPTOTIC_SIGNALING_PATHWAY, GO :0097190; GC response score: GOBP_CELLULAR_RESPONSE_TO_GLUCOCORTICOID_STIMULUS, GO:0071385; Inflammation score: GOBP_ACUTE_INFLAMMATORY_RESPONSE, GO:0002526; B-cell differentiation score: GOBP_B_CELL_DIFFERENTIATION, GO:0030183) were sourced from the Gene Set Enrichment Analysis (GSEA, www.gsea-msigdb.org/gsea/msigdb/collections.jsp#H). The UCell R package was utilized to estimate gene signature enrichment scores (UCell scores) in single-cell and ST datasets by employing the rank-based enrichment scoring on the count matrix derived from scRNA-seq data to evaluate per-cell enrichment for each gene set.

**Correlation Analysis**

Correlation analysis was performed at the donor level to avoid pseudo-replication from ROI/bin measurements. Briefly, metadata (Fib_myo_subset@meta.data) were filtered to retain observations with non-missing score1, score2, and donor identifier (orig.ident). For each donor and group, donor-level values were computed as the mean of ROI/bin-level scores (score1 and score2); if ROI-level weights were available, weighted means could be substituted, but unweighted means were used herein. The association between donor-level score1 and score2 was assessed using Spearman’s rank correlation (two-sided; exact = FALSE), reporting Spearman’s ρ and p-values. Confidence intervals for ρ were estimated using nonparametric percentile bootstrap with 1,000 resamples implemented in the boot package (set.seed(123)). Unless otherwise specified, analyses were performed on all donors; subset analyses (e.g., OP only, or GC_sensitive within OP) were conducted by filtering the corresponding groups prior to aggregation.

**GC-sensitive Spot Definition**

We defined “GC-sensitive” cells using per-sample percentile thresholds to ensure cross-sample comparability despite distributional shifts due to sequencing depth and batch. Specifically, we labelled cells above the 75th percentile of GC_score within each sample (Top25%) as GC-sensitive; sensitivity analyses used alternative percentiles (Top10/20/30) and a per-sample z-score threshold (e.g., z ≥ 0.7). For each definition, we computed sample-level effects as the mean difference between GC-sensitive and GC-low cells (GC_score_diff and Apoptosis_diff), summarized by group (OP, IPF) with 95% CIs (mean ± 1.96 × SE). We additionally estimated distribution-free 95% CIs via nonparametric bootstrap with 1,000 replicates at the sample level. Concordance between percentile- and z-threshold definitions was assessed by per-sample sensitive proportions and Spearman correlations were used to quantify associations between GC score and Apoptosis score (overall and by group). All thresholds and analyses were applied within-sample to preserve comparability across samples.

To visualise the spatial distribution density of GC_sensitive spots within each ROI, we estimate a per-sample sensitive density field via a 2D kernel density over spot coordinates. For each sample s, with GC_sensitive spot coordinates $(x_{i},y_{i})$, the sensitive density field $D_{s}(x,y)$is computed as $D_{s}(x,y)=\sum_{i} K_{h}(x-x_{i},y-y_{i})$, where $K_{h}$is a Gaussian kernel with bandwidth $h$determined by the defaults of MASS::kde2d. We render this field as filled polygons and dashed contours using stat_density_2d to highlight areas of higher clustering. To enable cross-sample comparability, legend limits reflect $\min(D_{s})$to $\max(D_{s})$aggregated across all samples, so density levels are harmonized and differences in spatial concentration can be interpreted consistently. Additionally, GC_sensitive spots are overlaid as distinct markers (black triangles) to distinguish point locations from the continuous density field, and the GC_score of all fibroblast_spots is shown as a separate gradient to differentiate transcriptional intensity from spatial clustering.

**MRC-5 Cell Culture and Stimulation**

MRC-5 human foetal lung fibroblasts (CL-0161, Procell, Wuhan, China) were cultured in Minimum Essential Medium (MEM; Gibco, Thermo Fisher Scientific, USA) supplemented with 10% foetal bovine serum (FBS), streptomycin at 37 °C in a humidified atmosphere containing 5% CO₂. For stimulation experiments, cells were seeded into appropriate culture plates and allowed to adhere overnight, then treated with recombinant human TGF-β1 (2.5 ng/mL; HY-P70433, MedChemExpress, USA) for 48h. Cells in another treatment group were treated with Dexamethasone phosphate (10^-6^M; HY-B1829A, MedChemExpress, USA) for 48h. Following intervention, fluorescence signals were examined by fluorescence microscopy, and the expression of relevant proteins was assessed by western blot analysis.

**Isolation and Characterization of Human Peripheral Blood B Cells**

Peripheral blood (50 mL) was obtained from healthy adult donors into EDTA-anticoagulated tubes and processed within 2 h, in accordance with institutional ethical approval and written informed consent. Peripheral blood mononuclear cells (PBMCs) were isolated using a human PBMC separation solution (Solarbio, P8610) according to the manufacturer’s instructions, by layering undiluted whole blood onto the separation medium and collecting the mononuclear cell layer after density-gradient centrifugation. The mononuclear cell layer was collected, washed, and resuspended in autoMACS™ Running Buffer (Miltenyi Biotec, 130-091-221-1). Human B cells were subsequently purified using the Pan B Cell Isolation Kit (Miltenyi Biotec, 130-101-638) according to the manufacturer’s instructions for negative selection. PBMCs were incubated with the antibody cocktail and magnetic beads, and the labelled suspension was applied to LS Separation Columns (Miltenyi Biotec, 130-042-401) placed on a MidiMACS Separator with MACS MultiStand (Miltenyi Biotec, 130-042-302 and 130-042-303). The flow-through containing negatively selected B cells was collected, and columns were washed with running buffer to optimize cell recovery.

An aliquot of isolated cells was stained with FITC-conjugated anti-human CD19 (clone HIB19; BD Biosciences, 555412) for 20 min at 4°C in the dark, washed with PBS containing 2% FBS, and analysed by flow cytometry. Data were acquired using a standard gating strategy (singlets → live cells → lymphocytes by FSC/SSC → CD19⁺), and B cell purity was reported as the percentage of CD19⁺ events within the lymphocyte gate, with appropriate controls included as required. Purified B cells were counted, and cells were resuspended in the designated culture medium for in vitro co-culture experiments.

**B Cell–Conditioned Medium Co-culture and Scratch Wound Assay in MRC-5 Fibroblasts**

Human B cells isolated as described above were cultured in RPMI-1640 medium (Gibco, Thermo Fisher Scientific, USA) supplemented with 10% heat-inactivated foetal bovine serum (FBS), at 37°C in a humidified atmosphere of 5% CO₂. Cells were divided into a resting group (no stimulation) and an activated group. For activation, B cells were stimulated with recombinant human IL-4 (10 ng/mL) and recombinant human CD40L (50 ng/mL) (all from MedChemExpress, MCE, China) for 48 h. After incubation, cultures were centrifuged, and the supernatants were collected and clarified to obtain resting or activated B cell–conditioned media. Each type of B cell–conditioned medium was then mixed 1:1 (v/v) with standard MRC-5 culture medium and applied to MRC-5 monolayers to establish the Rest B cell and Active B cell co-culture groups, respectively.

In subsets of the Active B cell co-cultures, the MIF inhibitor MIF098 (MedChemExpress, HY-147390) was added to the B cell–conditioned medium (Active B cell + MIF098 group). In parallel, some MRC-5 cultures were pretreated for 30 min with the anti-CD74 monoclonal antibody Milatuzumab (MedChemExpress, HY-P99731) before addition of Active B cell–conditioned medium (Active B cell + Milatuzumab group). MRC-5 cells were cultured in Active B medium and treated with recombinant human TGF-β1 (2.5 ng/mL; MedChemExpress, HY-P70433) to evaluate B-cell responses to TGF-β1–stimulated myofibroblasts (Active B cell + TGF-β1 group). MRC-5 cells cultured in standard medium alone served as the baseline control group, and an additional control condition consisted of MRC-5 cells cultured with recombinant human MIF (MedChemExpress, HY-P70288) to mimic an activated B cell–like MIF-rich environment for comparison with the B cell–conditioned medium groups.

After 48 h of the indicated treatments, a scratch wound assay was performed. Briefly, a linear scratch was created in the MRC-5 monolayer using a sterile pipette tip, detached cells were removed by washing with PBS, and cultures were returned to their respective media. Cell migration into the wound area was monitored and imaged at 0, 12, 24, 36, and 48 h using microscopy, and cell migration distance at each time point was analysed among the seven treatment groups using one-way ANOVA followed by Tukey’s multiple-comparison post hoc test.

**Immunofluorescence Analysis**

Immunofluorescence analysis was performed in cultured cells and human lung paraffin sections to evaluate fibroblast activation, glucocorticoid receptor nuclear translocation, apoptosis, and lineage-specific markers. For MRC-5 fibroblasts grown on sterile glass coverslips in 24-well plates, cells were washed with PBS, fixed with 4% paraformaldehyde for 15 minutes at room temperature, permeabilized with 0.1% Triton X-100 in PBS for 10 minutes, and blocked with 5% bovine serum albumin in PBS for 1 hour at room temperature. Primary antibodies diluted in blocking buffer were applied overnight at 4°C, including rabbit anti–ACTA2 (Proteintech, 14395-1-AP, 1:200), mouse anti–fibronectin (Proteintech, 66042-1-Ig, 1:200), rabbit anti–NR3C1/glucocorticoid receptor (Proteintech, 24050-1-AP, 1:200), mouse anti–Caspase-3 (Servicebio, GB12532, 1:200), and rabbit anti–Cleaved Caspase-3 (Servicebio, GB11532, 1:200), and rabbit anti–CD74 (Servicebio, GB115427, 1:200).The following day, cells were washed with PBS and incubated for 1 hour at room temperature in the dark with species-appropriate fluorophore-conjugated secondary antibodies, followed by nuclear counterstaining with DAPI for 5 to 10 minutes. Coverslips were mounted with antifade medium and imaged using confocal microscopy. ACTA2 and fibronectin were used to evaluate myofibroblast differentiation and extracellular matrix deposition, NR3C1 nuclear translocation was assessed by co-localisation with DAPI-stained nuclei, and Caspase-3 and Cleaved Caspase-3 signals were to assess apoptosis, and CD74 staining was used to assess activation of the MIF–CD74 signalling axis. For suspension B cells, cells were collected and washed with PBS, deposited onto poly-L-lysine–coated glass slides by gentle centrifugation, air-dried, fixed with 4% paraformaldehyde, permeabilized with 0.1% Triton X-100, and blocked with 5% BSA for 1 hour, then incubated with rabbit anti–MIF (Proteintech, 83199-2-RR, 1:200) and mouse anti–IgD (Proteintech, 67538-1-Ig, 1:200), mouse anti–IgM (Abcam, ab200541, 1:200) followed by appropriate fluorophore-conjugated secondary antibodies and DAPI staining. Slides were mounted with antifade medium and imaged by confocal microscopy.

Human lung tissue was processed as paraffin sections after fixation in paraformaldehyde for 24 hours, dehydration through graded ethanol, paraffin embedding, and sectioning at 4 to 6 μm thickness. Antigen retrieval was carried out using citrate buffer at pH 6.0 with microwave heating for 10 minutes, and sections were permeabilized with 0.1% Triton X-100, followed by blocking with 5% foetal bovine serum in PBS for 30 minutes. Primary antibodies diluted in blocking buffer were incubated overnight at 4°C and included rabbit anti–ACTA2 (Proteintech, 14395-1-AP, 1:200), rabbit anti–NR3C1/glucocorticoid receptor (Proteintech, 24050-1-AP, 1:200), rabbit anti–SFTPC (Proteintech, 10774-1-AP, 1:200), mouse anti–Collagen I (Proteintech, 66761-1-Ig, 1:200), mouse anti–CD19 (Immunoway, YM4248, 1:200), rabbit anti–Cytokeratin 5 (KRT5, Immunoway, YM8114, 1:200), rabbit anti–CD74 (Servicebio, GB115427, 1:200), rabbit anti–MIF (Proteintech, 83199-2-RR, 1:200), mouse anti–IgD (Proteintech, 67538-1-Ig, 1:200), rabbit anti–IgJ (Abcam, ab105229, 1:200), mouse anti–Cleaved PARP1 (Proteintech, 60555-1-Ig, 1:200), mouse anti–Caspase-3 (Servicebio, GB12532, 1:200), rabbit anti–Cytokeratin 17 (KRT17, Immunoway, YM6165, 1:200), rabbit anti–SCGB1A1 (Proteintech, 26909-1-AP, 1:200), mouse anti–LTF (Proteintech, 68509-1-Ig, 1:200), mouse anti–CD68 (Immunoway, YM3050, 1:200), rabbit anti–FABP4 (Servicebio, GB115466, 1:200), mouse anti–SPP1 (Abcam, ab166709, 1:200), rabbit anti–FCN1 (Servicebio, GB113616, 1:1500). Fluorophore-conjugated secondary antibodies were applied for 1 hour in the dark, including Goat Anti-Rabbit IgG H&L Alexa Fluor 488 (ab150077) and 594 (ab150080), Goat Anti-Mouse IgG H&L Alexa Fluor 488 (ab150113), 594 (ab150116), and 647 (ab150115), and Goat Anti-Rat IgG H&L Alexa Fluor 488 (ab150157) and 594 (ab150160), followed by DAPI nuclear counterstaining at 1 μg/mL for 5 minutes. Slides were mounted using antifade medium and imaged by fluorescence or confocal microscopy. Imaging parameters were kept consistent across experiments and exposure settings were controlled to avoid saturation, representative fields were selected using pre-defined anatomical and cellular criteria, and co-localisation and signal quantification were performed using standard ROI-based workflows.

**Western Blot Analysis of Protein Expression in MRC-5 Cells**

Total protein was extracted from MRC-5 cells using RIPA lysis buffer supplemented with protease inhibitors on ice. Cell lysates were clarified by centrifugation at 12,000 × g for 15 min at 4°C, and protein concentrations were determined using a BCA protein assay. Equal amounts of protein (20–40 μg per lane) were mixed with loading buffer, boiled for 5 min, separated by SDS–PAGE on 10% polyacrylamide gels, and electrotransferred onto PVDF membranes. Membranes were blocked with 5% non-fat dry milk in TBS containing 0.1% Tween-20 (TBST) for 1 h at room temperature and then incubated overnight at 4°C with primary antibodies diluted in blocking buffer: rabbit anti–ACTA2 (Proteintech, 14395-1-AP; 1:1000), mouse anti-fibronectin (Proteintech, 66042-1-Ig; 1:1000), rabbit anti-NR3C1 (Proteintech, 24050-1-AP; 1:1000), rabbit anti-CTHRC1 (Proteintech, 16534-1-AP; 1:1000), and rabbit anti–β-actin (Servicebio, GB15001; 1:1000) as a loading control. After washing with TBST, membranes were incubated with appropriate HRP-conjugated secondary antibodies for 1 h at room temperature. Protein bands were visualised using an enhanced chemiluminescence detection system and imaged with a digital imaging system. Densitometric analysis was performed using ImageJ software, and target protein expression levels were normalised to β-actin.

**Statistical Analysis**

All statistical analyses were performed using R (version 4.3.0) and GraphPad Prism (version 9). Statistical methods for each analysis are detailed in their respective method sections. No statistical method was used to predetermine sample size. For human samples, it was maximized based on availability, tissue quality, cost and throughput, and the number of donors aligns with similar spatial transcriptomics studies (10). The experiments were not randomised. The Investigators were blinded to allocation during experiments and outcome assessment.

**Reference**

1. Hao Y, Stuart T, Kowalski MH, Choudhary S, Hoffman P, Hartman A, Srivastava A, Molla G, Madad S, Fernandez-Granda C, Satija R. Dictionary learning for integrative, multimodal and scalable single-cell analysis. Nat Biotechnol 2024; 42: 293-304.

2. McGinnis CS, Murrow LM, Gartner ZJ. DoubletFinder: Doublet Detection in Single-Cell RNA Sequencing Data Using Artificial Nearest Neighbors. Cell Syst 2019; 8: 329-337.e324.

3. Korsunsky I, Millard N, Fan J, Slowikowski K, Zhang F, Wei K, Baglaenko Y, Brenner M, Loh PR, Raychaudhuri S. Fast, sensitive and accurate integration of single-cell data with Harmony. Nat Methods 2019; 16: 1289-1296.

4. Street K, Risso D, Fletcher RB, Das D, Ngai J, Yosef N, Purdom E, Dudoit S. Slingshot: cell lineage and pseudotime inference for single-cell transcriptomics. BMC Genomics 2018; 19: 477.

5. Lingampally A, Truchi M, Mauduit O, Delcroix V, Vasquez-Pacheco E, Gautier-Isola M, Chu X, Khadim A, Chao CM, Zabihi M, Taghizadeh S, Rivetti S, Marega M, Moiseenko A, Hadzic S, Vazquez-Armendariz AI, Herold S, Gunther S, Millar-Buchner P, Koepke J, Samakovlis C, Wilhelm J, Bartkuhn M, Braun T, Weissmann N, Zhang J, Wygrecka M, Makarenkova HP, Gunther A, Seeger W, Chen C, El Agha E, Mari B, Bellusci S. Evidence for a lipofibroblast-to-Cthrc1 (+) myofibroblast reversible switch during the development and resolution of lung fibrosis in young mice. Eur Respir J 2025; 65.

6. Yin Y, Koenitzer JR, Patra D, Dietmann S, Bayguinov P, Hagan AS, Ornitz DM. Identification of a myofibroblast differentiation programme during neonatal lung development. Development 2024; 151.

7. Jin S, Guerrero-Juarez CF, Zhang L, Chang I, Ramos R, Kuan CH, Myung P, Plikus MV, Nie Q. Inference and analysis of cell-cell communication using CellChat. Nat Commun 2021; 12: 1088.

8. Cable DM, Murray E, Zou LS, Goeva A, Macosko EZ, Chen F, Irizarry RA. Robust decomposition of cell type mixtures in spatial transcriptomics. Nat Biotechnol 2022; 40: 517-526.

9. Larsson L, Franzén L, Ståhl PL, Lundeberg J. Semla: a versatile toolkit for spatially resolved transcriptomics analysis and visualization. Bioinformatics 2023; 39.

10. Franzen L, Olsson Lindvall M, Huhn M, Ptasinski V, Setyo L, Keith BP, Collin A, Oag S, Volckaert T, Borde A, Lundeberg J, Lindgren J, Belfield G, Jackson S, Ollerstam A, Stamou M, Stahl PL, Hornberg JJ. Mapping spatially resolved transcriptomes in human and mouse pulmonary fibrosis. Nat Genet 2024.
